# Application of nanotechnology in the early diagnosis and comprehensive treatment of gastrointestinal cancer

**DOI:** 10.1186/s12951-022-01613-4

**Published:** 2022-09-15

**Authors:** Shenghe Deng, Junnan Gu, Zhenxing Jiang, Yinghao Cao, Fuwei Mao, Yifan Xue, Jun Wang, Kun Dai, Le Qin, Ke Liu, Ke Wu, Qianyuan He, Kailin Cai

**Affiliations:** 1 Department of Gastrointestinal Surgery, Union Hospital, Tongji Medical College, Huazhong University of Science and Technology, Wuhan, 430022 Hubei China; 2 Department of Neonatal Intensive Care Unit, Tongji Hospital, Tongji Medical College, Huazhong University of Science and Technology, Wuhan, 430022 Hubei China

**Keywords:** Gastrointestinal cancer, Nanotechnology, Nanoparticles, Early diagnosis, Targeted therapy, Surgical navigation, Tumour imaging

## Abstract

Gastrointestinal cancer (GIC) is a common malignant tumour of the digestive system that seriously threatens human health. Due to the unique organ structure of the gastrointestinal tract, endoscopic and MRI diagnoses of GIC in the clinic share the problem of low sensitivity. The ineffectiveness of drugs and high recurrence rates in surgical and drug therapies are the main factors that impact the curative effect in GIC patients. Therefore, there is an urgent need to improve diagnostic accuracies and treatment efficiencies. Nanotechnology is widely used in the diagnosis and treatment of GIC by virtue of its unique size advantages and extensive modifiability. In the diagnosis and treatment of clinical GIC, surface-enhanced Raman scattering (SERS) nanoparticles, electrochemical nanobiosensors and magnetic nanoparticles, intraoperative imaging nanoparticles, drug delivery systems and other multifunctional nanoparticles have successfully improved the diagnosis and treatment of GIC. It is important to further improve the coordinated development of nanotechnology and GIC diagnosis and treatment. Herein, starting from the clinical diagnosis and treatment of GIC, this review summarizes which nanotechnologies have been applied in clinical diagnosis and treatment of GIC in recent years, and which cannot be applied in clinical practice. We also point out which challenges must be overcome by nanotechnology in the development of the clinical diagnosis and treatment of GIC and discuss how to quickly and safely combine the latest nanotechnology developed in the laboratory with clinical applications. Finally, we hope that this review can provide valuable reference information for researchers who are conducting cross-research on GIC and nanotechnology.

**Graphical Abstract:**

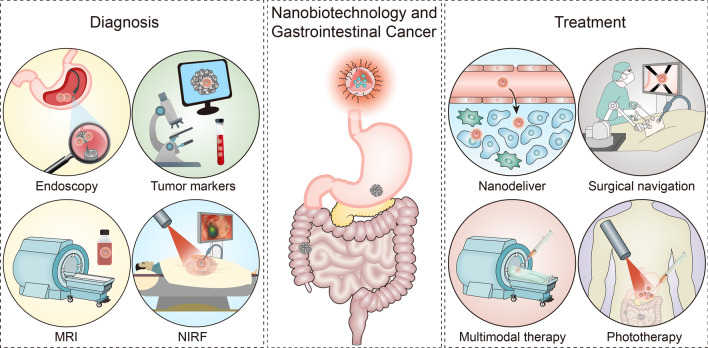

## Introduction

According to the latest cancer statistics from the American Cancer Society, the proportions of new cases and deaths of gastrointestinal malignancies among all tumour cases in 2022 are 17.9% and 28.3%, respectively [1]. Gastric cancer (GC) and colorectal cancer (CRC) are two types of malignant gastrointestinal tumours with the highest incidence. The high invasiveness and heterogeneity of GC and CRC are the main causes of death in patients with malignant gastrointestinal tumours [2–5]. The mean 5-year overall survival rate for CRC and GC is approximately 60%, and it is worth noting that the survival rates decrease significantly after metastasis development [6–8]. Therefore, prolonging the survival times of patients with gastrointestinal cancer (GIC) is an urgent problem to be solved.

According to clinical experience, most patients with early gastric cancer have no obvious symptoms, while a few have nausea, vomiting or upper gastrointestinal symptoms that are similar to ulcer disease. For patients with advanced gastric cancer, there are often obvious symptoms in the upper gastrointestinal tract, such as epigastric discomfort, fullness after eating, increased abdominal pain, decreased appetite, and fatigue [9]. Similarly, most patients with early colorectal cancer are asymptomatic, or the symptoms are not obvious, and they only feel discomfort, indigestion and have faecal occult blood. As tumours develop, symptoms gradually appear, including changes in bowel habits, abdominal pain, blood in the stool, abdominal masses, and intestinal obstructions, with or without systemic symptoms such as anaemia, fever, and weight loss [10]. In summary, because the early symptoms of patients with gastric and colon cancer are not obvious, most patients are diagnosed in the middle and late stages, which will seriously affect the therapeutic effects for patients. Early detection and early treatment can greatly improve the cure rate and reduce mortality to improve the quality of life of patients and prolonging lives is crucial. Therefore, early diagnosis and treatment of patients with digestive tract tumours are very important.

In the current clinical diagnoses of GIC, clinicians make qualitative, localization and staging diagnoses of patients based on the results of laboratory examinations, imaging examinations and pathological examinations. Among them, imaging diagnoses are the most important diagnostic technique, and they can help doctors identify the presence of metastatic lesions before clinical staging and treatment, which include endoscopy, magnetic resonance imaging (MRI), computed tomography (CT) and positron emission tomography (PET) [11–17]. Early and precise diagnoses can provide essential reference information for treating GIC. For small early-stage lesions, surgical resection and phototherapy might achieve good therapeutic efficacy. Phototherapy also has a promising future for treating CRC because of its low invasiveness and toxicity [18]. Photodynamic therapy (PDT) and photothermal therapy (PTT) are two standard phototherapy methods. For photothermal therapy, light at a specific wavelength irradiates photothermal agents, which heat up and kill tumour cells. In photodynamic therapy, photosensitizers can produce large amounts of reactive oxygen species that can kill tumour cells under specific types of light exposure [19]. For advanced gastrointestinal malignancies without distant metastases, accurate clinical staging and evaluations are required before surgical treatment or preoperative combined neoadjuvant chemoradiotherapy. For GIC with distant metastases, individualized treatment plans need to be formulated after comprehensive evaluations based on the locations, numbers, and sizes of metastatic lesions [20, 21]. Although new treatment methods, such as immunotherapy and biotherapy, have made promising breakthroughs in their curative effects, obtaining diagnostic imaging information is still necessary for clinicians to choose an appropriate treatment plan for gastrointestinal tumours. We can maximize the therapeutic effect only by selecting the most suitable treatment scheme that is based on accurate diagnostic information [22–27].

Currently, the main problem when diagnosing GIC is that technical methods are limited in their sensitivity for diagnosing small lesions and tumour markers in the early stage or after radiotherapy and chemotherapy. Endoscopy is one of the most widely used invasive examinations and can be used to directly observe tumours in the gastrointestinal tract. Convenience and speed are the main advantages of endoscopy. However, its disadvantages are also clear: poor recognition of small, early lesions and the inability to observe small lesions that are below the picture resolution [28, 29]. If clinicians need to determine whether a patient has other small lesions, they can rely only on technical means such as MRI or CT to obtain the necessary information. However, because the gastrointestinal tract is different from other organs, it exhibits the phenomenon of involuntary movement, resulting in the overall sensitivity of MRI, CT and PET in GIC detection being relatively low [30, 31]. In the treatment of GIC, despite the use of biological agents combined with radiotherapy and chemotherapy and the combination of multiple therapeutic modalities to inhibit tumour recurrence and progression, the weak targeting and affinity of drugs lead to low therapeutic efficiencies and serious side effects for patients [32, 33]. Therefore, there is an urgent need to develop other techniques that can overcome the challenges presented by the low sensitivity of imaging results and low drug utilization caused by gastrointestinal autonomic movements.

**Fig. 1:**
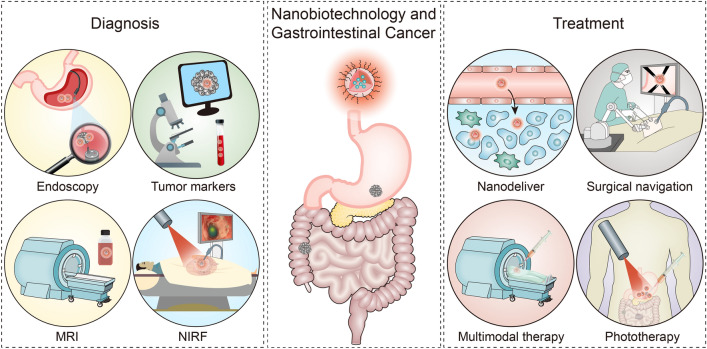
The application of nanotechnology in the diagnosis and treatment of gastrointestinal cancer

In recent years, with new nanoparticles being developed to diagnose and treat cancer, there have been unprecedented developments in the field of nanomedicine (Fig. 1). In the early diagnosis and screening of GIC, new biosensors based on nanotechnology can improve the sensitivity of clinical diagnoses, and some pathological tissues or organs can be detected earlier and more accurately [34]. For example, biocompatible nanoprobes for molecular and cellular imaging, integrated nanodevices for early cancer screening, and immuno-microfluidic chips with semiconductor quantum dots (QDs) enable precise detection of human-associated tumour markers, which can guide more effective treatments of GIC [35–37]. However, even if other small tumour lesions in a patient's body are found, improving drug solubility and utilization efficiency and addressing drug resistance are still challenges that must be overcome in the clinical treatment of GIC. Using nanotechnology to construct a drug delivery system is an effective method to improve drug utilization, and its biggest advantage is its large specific surface area and flexible modification capability. Modifying nanoparticle surfaces can more effectively target tumour tissues, thus increasing the effective concentrations of drugs in tumour regions while reducing the side effects of chemotherapeutic drugs and improving the effectiveness of chemotherapeutic drug therapy [38–42]. Their large functional surface areas of nanoparticles allow them to bind, absorb, and carry other compounds, such as small-molecule drugs, DNA, RNA, proteins, and probes. In addition, their adjustable size, shape, and surface properties give them high stability, high carrier capacity, the ability to absorb hydrophilic and hydrophobic substances, and they are compatible with different drug delivery pathways, making them highly attractive in many aspects of medicine [43]. Additionally, we can also construct nanoparticles with diagnostic and therapeutic functions, which are well designed for more specific and personalized disease management by combining diagnostic and therapeutic capabilities into one single biocompatible and biodegradable nanoparticle, which can improve the efficacy for gastrointestinal tumours and have attracted extensive attention [44]. Due to their high specific surface areas and surface and interfacial effects, nanoparticles have a natural advantage as drug carriers, which can be used not only for imaging but also in combination with bioactive agents to benefit cancer therapy. Currently, a variety of theranostic nanoparticles are available for drug delivery, such as metals, polymer-based NPs, liposomes and others [45]. To further ameliorate the influences of gastrointestinal autonomic movements on the early diagnosis and treatment of GIC, this review comprehensively introduces the applications of nanotechnology in the early diagnosis and treatment of GIC and the clinical challenges for GIC treatment and discusses how to promote the clinical use of nanoplatforms built in the laboratory as soon as possible. The purpose of this review is to establish a bridge between clinical GIC therapy and laboratory nanotechnology and to provide guidance for the development of nanomedicine in the field of GIC therapy.

## Application of nanotechnology in the early diagnosis of GIC

Based on many years of clinical data, patients with GIC who are diagnosed with early-stage tumours and precancerous lesions have better prognoses, lower mortality rates, and longer survival times than patients with progressive disease [46]. The current clinical applications of early-diagnosis methods for GIC mainly include endoscopy, tumour markers, MRI, CT, PET and NIRF detection. With the development of science and technology and increased understanding of the abovementioned diagnostic mechanisms, researchers have found that these diagnostic methods have room for improvement and enhancement. For example, the use of capsule endoscopy can reduce discomfort in patients due to invasive examinations and decrease the chance of inflammation. Injecting contrast agents prior to MRI imaging can help to improve its diagnostic sensitivity. In recent years, many researchers have combined nanotechnology with the abovementioned imaging technologies so that the sensitivity of these technologies has been further improved. The applications of nanotechnology to the early diagnosis and therapeutic imaging of gastrointestinal cancer will be introduced below, and the transition from laboratory research to clinical application will be pointed out.

### Gastrointestinal endoscopy

Endoscopy is a valuable examination technique used in gastrointestinal cancer screening programs to detect and treat early precursor lesions and diagnose malignancies quickly. Endoscopic white light imaging can observe the mucosal morphology of gastrointestinal tract tissues in real time, while tissue biopsies can be obtained from highly suspected lesions to further aid diagnosis [47, 48]. However, endoscopy also faces great challenges for early diagnoses of GIC. The recognition of early microscopic lesions was poor, and microscopic lesions below the image resolution could not be observed [20, 21]. At the same time, the performance of endoscopy varies greatly among different endoscopists, resulting in a missed diagnosis rate of early gastrointestinal cancer of 20–40% [49]. In addition, endoscopic diagnosis is subjective and operator dependent and varies greatly with experience, reducing the detection rates of gastrointestinal cancer and precursor lesions. Endoscopy are semi-invasive procedures that often cause discomfort and fear in patients, thus affecting early GIC examinations [50, 51]. Therefore, as an indispensable diagnostic technique for early screening of gastrointestinal diseases, improving the comfort and accuracy of endoscopy is the main research direction in the future.

Clinically, some patients will choose anaesthesia to avoid the discomfort caused by endoscopy, but this cannot solve the fundamental problem. Especially with a small intestinal endoscope, carefully observing pathological changes in the small intestine has been a difficult problem for clinicians. The small intestine is characterized by more bending, more peristalsis and an unstable position. In endoscopy, if the endoscope is not operated properly, it is easy to cause complications such as mucosal bleeding or perforation in the patient, and in more serious cases, endoscopy will cause life dangers to the patient. Nanotechnology has promising applications in endoscopy for haemostasis of peptic bleeding, prevention of plastic stent blockage, advanced capsule endoscopy, early cancer screening, and other endoscopic applications [52–54]. The first FDA-approved "smart pill", PillCam, a medical product created by the combination of plasma, nano- and digital technologies, can provide earlier and more accurate diagnoses of various gastrointestinal diseases, including cancer [55, 56]. The Pill Cam is an example of the clinical transformation of nanotechnology research and beneficial linkages with digital technologies, with nanotechnology being used to develop a biocompatible coating (wrapped around a capsule) that can resist strong stomach acid and digestive enzymes. Meanwhile, nanotechnology is used to create endoscope capsules at the molecular level, while digital technology allows images to be transmitted to computers via sensors attached to the abdomen to enable more accurate and earlier diagnoses of many diseases, including cancer. Capsule endoscopy is performed by ingesting a small (26 × 11 mm) disposable battery-powered pill containing a complementary metal oxide semiconductor camera, which provides a 156 field of view, variable depth of view (1–30 mm), and resolution of 0.1 mm. Four LEDs illuminate the intestinal lumen. Once swallowed by the patient, images of the gastrointestinal tract are captured, which are transmitted by radio frequency signals to an external portable storage unit and downloaded to a workstation [57]. The development and application of capsule endoscopy have solved many common problems that arise in traditional endoscopy. Since the advent of small intestinal capsule endoscopy, the applications of special oesophageal and colon capsule endoscopy have expanded to include investigations of upper and lower digestive tract diseases. Oesophageal capsule endoscopy can be used to diagnose oesophagitis, Barrett's oesophagus and varicose veins. Colon capsule endoscopy offers an alternative to traditional colonoscopy for symptomatic patients, and its possible role in colorectal cancer screening is a fascinating prospect. Current research has examined the possibility of controlling the movement of capsules and has developed capsules that can be used for tissue sampling and treatment [56]. Ankri et al. proposed a novel optical detection method specifically designed for colon cancer detection based on the well-known optical properties of immunoconjugated gold nanorods (GNRs). In a model of colon cancer implanted in a chick chorioallantoic membrane, this assay can differentiate between cancerous and normal tissues. The distance between the light source and intestinal wall and the background signal do not affect the detected signal and can decrease the number of false-positive and false-negative results and improve the identification of tumours and micrometastases [58]. In addition, with the development of various technologies, nanocapsule-molecular imaging and endoscopic optical biopsies have become key research projects. The endoscopic field intends to produce capsules with identification, anchoring and biological sensing abilities to achieve accurate pathological detections and diagnoses, such as using microgripper folding and grabbing tissue sample capsules [59]. In summary, application of nanotechnology in capsule endoscopy has greatly improved the accuracy of clinicians when diagnosing small intestinal tissue lesions, and with the progress in technology, the function of capsule endoscopy is also improving. For example, the use of multifunctional biocompatible nanomaterials wrapped in capsule endoscopes and the design of targeted capsule endoscopes or capsule endoscopes with biopsy functions are future research directions.

**Fig. 2:**
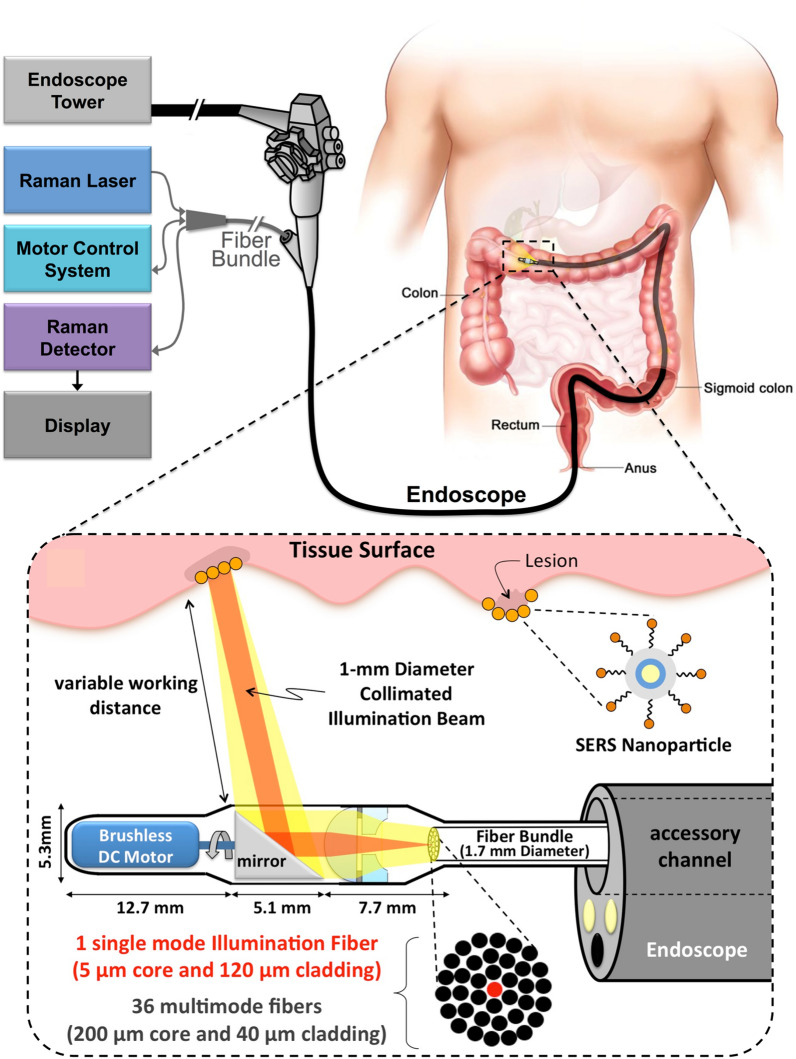
Schematic diagram of Raman imaging system used in parallel with SERS Nanoparticle and white light endoscope [63]

However, although capsule endoscopes can solve the comfort problem of endoscopes, it still provides the structural information obtained with white light. Although the disease detection ability has been greatly improved by white light endoscopy at present, it is easier for traditional white light endoscopy to miss small lesions due to the complex surface topography, folds of mucosa and peristalsis of viscera in the intestinal tract cavity. To address this need, Zavaleta et al. investigated and developed a unique accessory, noncontact, fibre-optic-based Raman spectroscopy device that has the potential to provide real-time, multiplexed functional information during routine endoscopies. The principles of operation are as follows: 1. local administration of tumour-targeted SERS nanoparticles for a specific region of interest during endoscopy, followed by washing of unbound SERS nanoparticles; 2. detection and quantification of bound targeted SERS nanoparticles using a Raman endoscopy device; 3. the pathological information of the patient is determined based on identifying the locations and types of bound SERS nanoparticles. SERS technology has obvious advantages in tumour imaging. SERS is a type of plasma effect, and molecules adsorbed on rough metal surfaces can produce high Raman scattering intensities, such as Au/Ag/Cu nanoparticles. The SERS effect can increase Raman signal intensities by up to 10^14^–10^15^ times. To improve the performance of SERS imaging, researchers have developed several SERS nanotags, such as gold nanostars, copper-based nanomaterials, and semiconducting quantum dots. To date, various SERS NPs (mostly gold- or silver-based) have been combined with various targeted ligands (such as polypeptides, proteins, antibodies, antibody fragments, DNA and adhesives) in molecular imaging applications. In addition, because SERS nanoparticles are nontoxic and metabolizable in vivo, they have been developed for medical applications of in vivo imaging, including cancer diagnosis, image-guided surgery and cancer treatment [60, 61]. Considering the advantages of SERS NPs and Raman endoscopy in imaging, some studies have also applied this technique to the early diagnosis of GIC to improve diagnosis rates. These study results indicate unparalleled sensitivity and multiplexing capabilities as well as efficient use of working distances of 1 to 10 mm, which allow the endoscopist to rapidly differentiate between normal and precancerous tissues and identify flat lesions that are often missed [62]. In addition, Garai et al. reported the design and in vivo demonstration of a miniature, noncontact, optomechanical Raman device that is capable of performing rapid circumferential scanning of the topologically complex luminal surfaces of hollow organs (e.g., colon and oesophagus) and generating quantitative images of the relative concentrations of SERS nanoparticles present. Simultaneous detection of the unique spectra of multiple SERS nanoparticle fingerprints provides unparalleled multiplexing capabilities. This new screening strategy has the potential to improve diagnoses and guide treatment through sensitive, quantitative molecular detections of small lesions and otherwise difficult to detect lesions under white-light endoscopy (Fig. 2) [63, 64]. Zavaleta et al. found that local application of SERS nanoparticles in the colon of mice appears to minimize their systemic distribution, thereby avoiding potential toxicity and supporting the clinical use of Raman spectroscopy as an endoscopic imaging tool [65]. Therefore, we suggest that SERS nanoparticles combined with conventional optical endoscopy may provide a more sensitive method to detect early gastrointestinal cancer than conventional tumour marker screening or optical endoscopy.

The continuous improvements in optical technology have greatly improved the resolution and contrast of endoscopes. At present, confocal laser microscopic endoscopes have the highest resolution. Confocal laser microendoscopy is a revolutionary endoscopic imaging technique that has been developed only in the past decade, which can obtain highly magnified cross-sectional images of gastrointestinal epithelium at the same time, and histological diagnoses can be obtained without biopsies or histopathological examinations. In clinical applications, Cellvizio is the first and only confocal laser microendoscope in the world (pCLE/nCLE) that is equipped with a probe and fine needle. It has been shown that Cellvizio can help doctors improve their decision-making confidence, reduce uncertainty and improve patient prognoses in the diagnosis and treatment of digestive tract, respiratory tract, urinary system diseases and many others. In the laboratory research and development processes, Du et al. used dye-labelled human heavy chain ferritin as an endoscopic imaging nanoprobe to identify the GC biomarker transferrin receptor 1, leading to specific early imaging of GC by using confocal laser microendoscopy, which is helpful in distinguishing between tumour and nontumor tissue and in visualizing endoscopic resections and tumour margin differentiations [66]. Li et al. labelled the anti-gastric cancer-specific molecular marker monoclonal antibody, MG7, with a fluorescent agent and imaged it in vivo in real time using confocal laser microendoscopy. They found that endoscopy combined with an MG7-labelled fluorescent agent helped detect MG7-Ag-positive tissue and facilitated early detection of GC [67].

With the rapid development of nanotechnology and other technologies, the applications of capsule endoscopy, laser confocal endoscopy and Raman spectroscopy combined with SERS nanoparticle endoscopy have greatly improved the accuracy of clinical diagnoses of malignant gastrointestinal tumours, especially the research and application of various multifunctional SERS nanoparticles. However, there are many factors restricting the application and clinical use of SERS nanoparticles in in vivo imaging. First, the penetration depth of SERS nanoparticles in tissues is limited, leading to a limited application of SERS imaging in deep tissues, the whole body or the intracranial system. Second, accurately and quickly capturing and analysing Raman scattering light from deep tissues also presents a challenge. Finally, the biosafety of SERS NPs is among the important factors that limit their clinical use. Therefore, from a future perspective, only the design and development of safe, stable and advanced SERS nanoparticles and corresponding high-precision Raman or other devices can expand the application of SERS nanoparticles to clinical diagnoses.

### Gastrointestinal malignancy markers

Detection of tumour markers is a noninvasive early detection method that has the advantages of convenient operation, easy sample collection and easy dynamic detection. It is also widely used in the early diagnosis of malignant gastrointestinal tumours in clinical applications. It also plays an important role in tumour staging, treatment guidance and prognosis judgements. At present, due to the variety of tumour markers and different detection methods, the main detection methods include radioimmunoassays, chemiluminescence immunoassays, enzyme-linked immunosorbent assays, immunosensor, proteomics, molecular biology, liquid biopsies and combined detections. They indicate the amounts of the molecule to be detected by using a signal produced by an enzyme, fluorescein, isotope or chemical luminescent agent labelled on an antigen or antibody.

Clinically, the common tumour markers of malignant gastrointestinal tumours mainly include embryonic antigens (e.g., AFP and CEA), glycoprotein antigens (e.g., CA-199, CA-125, CA15-3, CA72-4, CA-242, and CA-50) and protein antigens (CyFRA21-1 and Her 2). In addition, malignant gastrointestinal tumours can also cause epigenetic changes such as DNA methylation, specific histone modification, chromatin remodelling and noncoding RNA, such as colorectal cancer K-RAS mutation detection, microsatellite instability (MSI) screening, gastric cancer HER-2 detection, and gastrointestinal stromal tumour KIT protein detection, which can be considered important tumour markers [68–70]. These changes are used to detect and predict the prognosis of early malignant gastrointestinal tumours and to provide guidance for treatment. However, considering that different patients have different levels of secretion and expressions of tumour markers, in patients with different malignant gastrointestinal tumours due to different tumour stages and lesion sizes, for some patients with low expression levels, detection of tumour markers is likely to obtain results that would delay treatment [71, 72]. Therefore, it is of great significance for clinical diagnosis and treatment to improve the sensitivity of tumour marker detection and develop a new type of high-sensitivity tumour marker screening method for patients with malignant gastrointestinal tumours or other kinds of cancer.

**Fig. 3:**
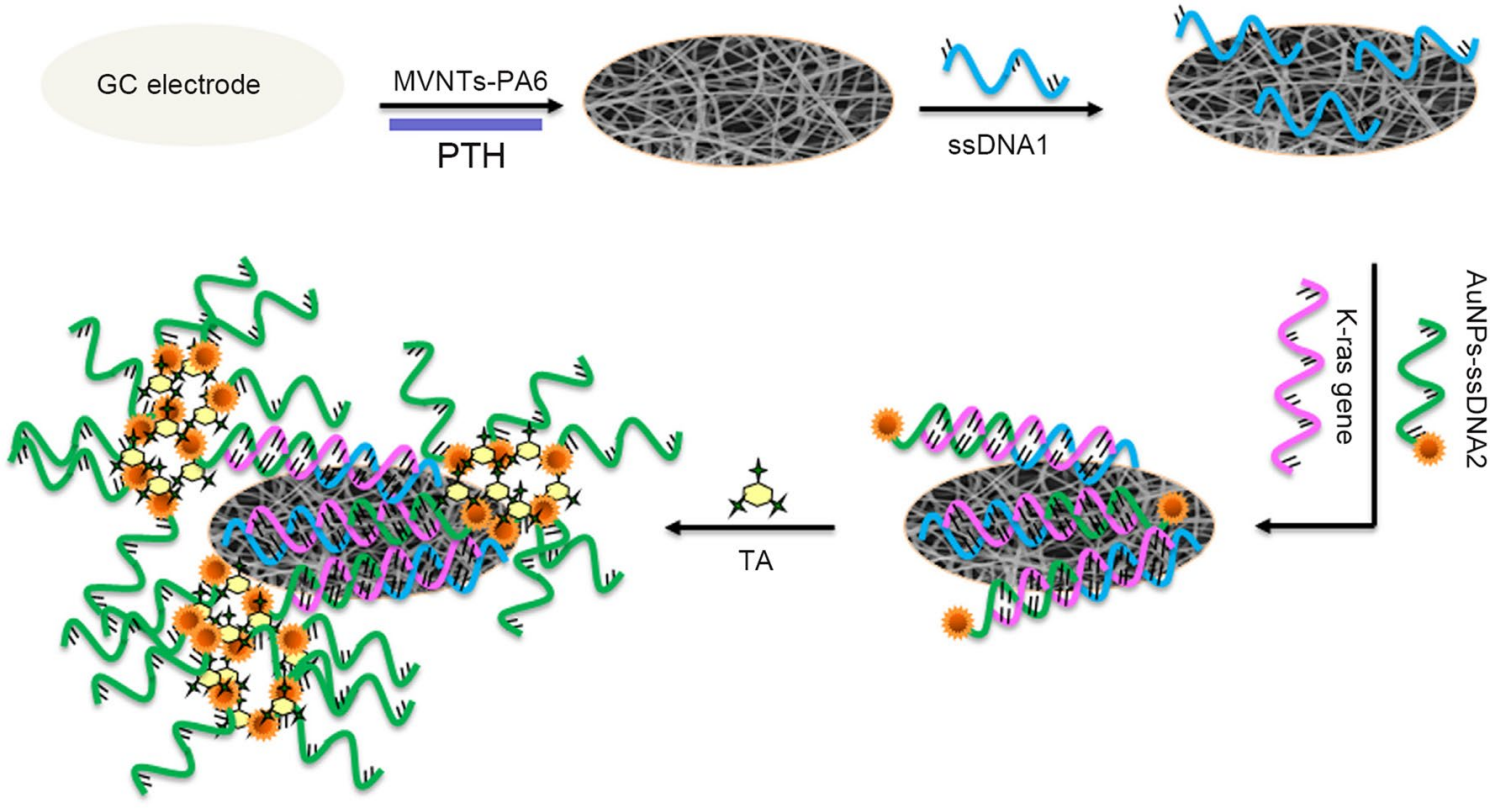
An electrochemical biosensor based on functional composite nanofibers detects k-RAS gene schematics through multi-signal amplification strategy [73]

In clinical fluid biopsies of gastrointestinal malignancies, tumour markers such as microRNA (miRNA), circulating tumour DNA (ctDNA), protein, exosome and circulating tumour cells (CTCs) exist at micron or nanometre levels, which promote the development of nanotechnology and application of tumour markers, especially plasma sensors based on nanomaterials. With the advantages of high sensitivity, good real-time sensing performance and good biocompatibility, these sensors have great application prospects in the clinical detection and diagnosis fields. The KRAS gene mutation at codon 12 is a common mutation in colon cancer. Wang et al. synthesized an electrochemical biosensor composed of functional composite nanofibres and used multiple signal amplification strategies to detect the CRC KRAS gene. Carboxylated multiwalled carbon nanotube (MWCNT)-doped nylon 6 (PA6) composite nanofibres (MWCNTs-PA6) were prepared by electrospinning to provide the nanoskeleton for the electropolymerization of thionine (TH). PTH poly(thionine) is used as a scaffold to fix single-stranded DNA1 (ssDNA1), which can significantly increase DNA attachment and hybridization sensitivity. The ssDNA1/K-RAS gene/gold nanoparticle-labelled ssDNA2 (AuNP-ssDNA2) sandwich structure was prepared by a hybridization reaction, and AuNPs provided good electrochemical signal transduction. The signal is further amplified by forming a network of thiocyanate/gold nanoparticles (TA/AuNPs). The results demonstrate that this nanocomposite has a good detection capability for K-RAS mutations (Fig. 3) [73]. They also used surface plasmon resonance (SPR) based on gold nanoparticle hybridization for sensitive miRNA diagnoses. The designed biosensor can detect human miRNA from cancer cells. It has great potential for quantitative miRNA detection in early clinical diagnoses and biomedical research [74]. MIR-106a is another potential tumour marker of colon cancer. Daneshpour et al. developed an ultrasensitive electrochemical nanobiosensor that uses a bispecific probe method and a gold-magnetic nanocomposite as a tracking tag to detect miR-106a. The results show that the nanobiosensor has significant selectivity, high specificity and good storage stability and can provide a promising application for the early clinical detection of GC [75]. In addition, Weigum et al. designed an optical biosensor platform based on the supramolecular interactions among graphene oxide nanoparticles and CRC tumour marker molecules. They found that structural modifications can change the binding strengths of the coupling motifs of molecular beacons that are fixed on the surface of nanocarriers, thus providing higher detection efficiency [76].

Detecting CTCs in the blood is also an effective way to screen malignant gastrointestinal tumours in the early stage. Tran et al. used a SiNW-FET-based intraoperative biosensing platform to detect the presence of circulating tumour cells in the blood of CRC cancer patients. The SiNW-FET sensing platform is not only superior to the current standard methods based on biopsy pathology in its sensitivity and speed but is also superior to the emerging clinical gold standard based on molecular detection, which demonstrates the potential of SiNW-FET in clinical cancer diagnosis. [77]. In addition, the biosensor based on silicon nanowires (SiNW) not only can detect circulating tumours in the blood but can also track the treatment process by determining the C-reactive protein concentrations in the blood of patients with malignant gastrointestinal tumours. What is most surprising is that Shehada et al. detected volatile organic compounds that are linked with gastric cancer conditions in exhaled breath samples and could discriminate them from environmental volatile organic compounds that are present in exhaled breath samples but are not related to gastric cancer progression through ultrasensitive molecular modified SiNWs [78].

In addition to obtaining biological information on gastrointestinal tumour markers in patients' blood or body fluids, nanotechnology can also help us to obtain information about the existence of tumour markers in tissue samples obtained from patients. In particular, quantum dot nanomaterials, which have high fluorescence quantum yields, anti-photobleaching stability, and size-controlled luminescence properties, have higher sensitivity and repeatability than traditional IHC. Therefore, they are suitable for photoelectrochemical tumour marker detection, especially for complex biological samples [79]. Xing et al. developed an imaging method based on coupling a glucose transporter 1 (Glut1) antibody with paramagnetic QDs. Diethylenetriaminepentaacetic acid dihydroxide (DTPA) was coupled with bovine serum albumin (BSA) and then chelated with Gd3 + to obtain the Gd-DTPA coupling fraction. Then, quantum dots were added to this conjugated fraction by sonication to obtain GdDTPA-BSA@QDs, and surface-targeted Glut1 polyclonal antibody (PcAb) adhered to GdDTPA-BSA@QDs. In vitro, the expression and distribution of Glut1 were studied by MRI scanning and confocal fluorescence imaging. MRI studies revealed that the nanoprobe was highly biocompatible, which improved the diagnostic accuracy [80].

Through the research results introduced above, it can be concluded that nanotechnology has the advantage of improving the sensitivity of tumour marker detection. In the future, the greatest challenge facing nanotechnology is how to successfully apply laboratory scientific research in clinical settings. In addition, whether for malignant gastrointestinal tumours or other tumours, creating nanomaterials that can provide simultaneous detection of multiple markers is another important research direction of nanotechnology in tumour marker diagnosis.

#### MRI

Magnetic resonance imaging is a commonly used diagnostic tool in clinics for cancer detection. Its principle is to use radio-frequency electromagnetic waves to excite nonzero spin nuclear matter in a static magnetic field to generate nuclear magnetic resonance and to collect magnetic resonance signals with an induction coil. Finally, the imaging method is processed according to a certain mathematical method. However, due to some limitations of the human abdomen (such as empty organs and involuntary movements), the imaging effect of MRI in the abdomen cannot be the same as that in muscle, bone or the central nervous system. Although a clinical MRI contrast agent can improve the signal-to-noise ratio and spatial resolution of MRI, because of its intravenous or subcutaneous injection, it is difficult to retain large amounts of MRI contrast agents in gastrointestinal tissues for a long time, and there are systemic nonspecific distributions, rapid clearance, poor pharmacokinetics and side effects, so MRI contrast agents cannot properly improve the imaging effect for malignant gastrointestinal tumours. Therefore, to construct malignant gastrointestinal tumour MRI contrast agents, the main method is to modify some targeted malignant gastrointestinal tumour groups in the contrast medium to form nanoparticles, which can be used in the early detection and tumour localization of GIC.

Paramagnetic ion complexes or superparamagnetic particles containing rare earth elements such as Gd (Gd^3+^) have the functions of shortening the T1 or T2 relaxation times, increasing the T1-weighted image signal intensities or reducing the T2-weighted image signal intensities, which have been used as nuclear magnetic resonance contrast agents in the clinic. To obtain a better imaging effect for GIC, Sun and Wang et al. designed solid lipid nanoparticles loaded with gadolinium (Gd), diethyltriamine pentaacetic acid (Gd-DTPA) and fluorescein octadecylamine isothiocyanate (FITC) imaging nanoparticle contrast agents. The results show that these can enhance the contrast of tumour sites and lead to long-term improvements in detecting CRC during MR colonography [81, 82]. Khantasup et al. developed a new contrast agent by coupling humanized anti-EpCAM antibodies with gadolinium chelate. The results show that the new contrast agent has a high targeting effect on colorectal cells and has the advantages of good stability, biocompatibility and imaging [83]. Shi et al. reported a tumour targeting and matrix metalloproteinase-2 (MMP-2)-activated nanoprobe (T-MAN) that was formed by covalent modification of GD-doped CuS micellar nanoparticles with crgd and MMP-2 cleavable fluorescent substrates. This study found that the modified nanoprobe has the advantage of dual-mode imaging to improve the selectivity of cancer treatment in vivo [84].

**Fig. 4:**
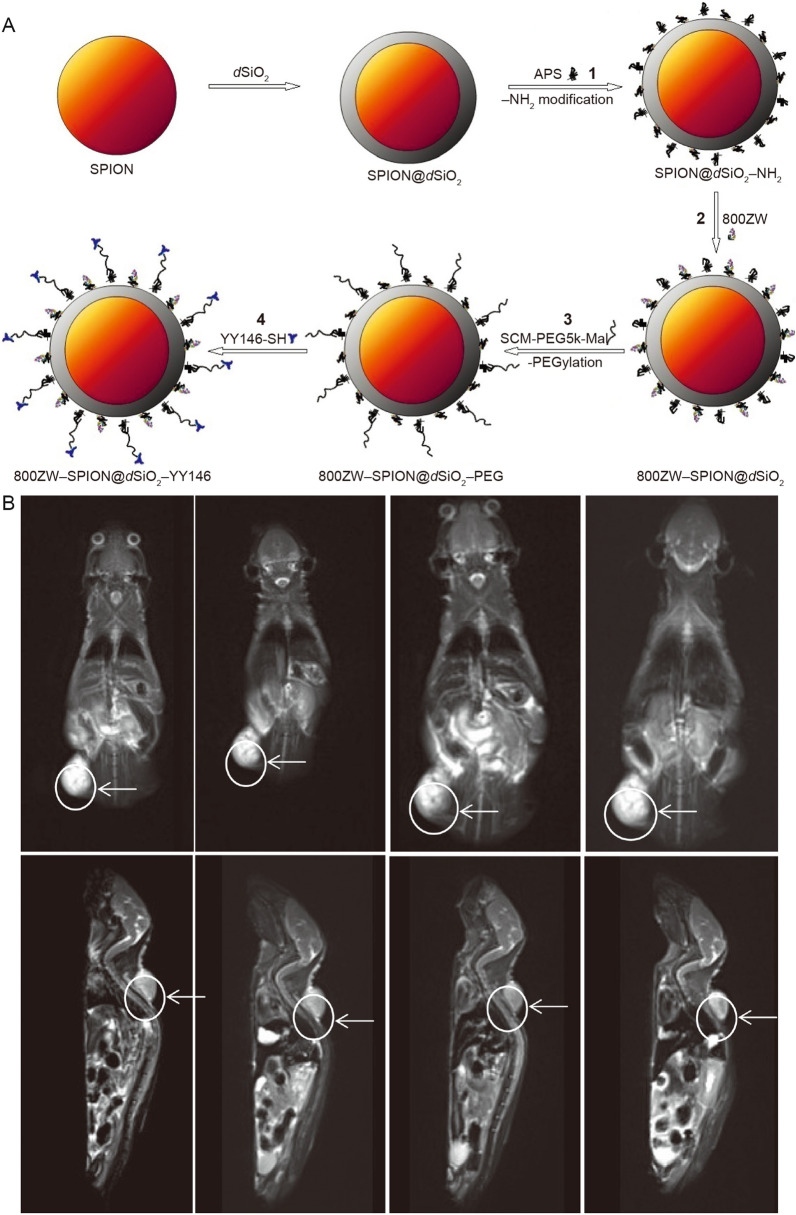
Nanotechnology improves MRI imaging efficiency. Schematic illustration of the synthesis of 800ZW–SPION@dSiO2–YY146 (**A**) and Series of images shows T2-weighted MR images obtained at a certain point in time on the 11.7 T system (**B**) [87]

Superparamagnetic iron oxide nanoparticles (SPION) are also widely used in MRI imaging of GIC [85, 86]. Wang et al. coated SPIONs with SiO2 as core–shell nanoparticles and labelled them with NIRF and an anti-CD146 monoclonal antibody for GIC imaging. The results show that this kind of functional nanomaterial is suitable for tumour imaging and treatment (Fig. 4) [87]. Yan et al. reported an MRI/optical bimodal molecular probe composed of polyethylene glycol-modified nano Fe_3_O_4_, the specific targeting cyclic peptide, GX1, and the near-infrared fluorescent dye, Cy5 5. It was found that nanoaggregates selectively in GC, which is expected to become a new probe for early diagnosis of GC [88]. Guo et al. developed a highly water-soluble biological nanoprobe by forming covalent bonds on the surfaces of iron oxide gold nanoclusters (Fe_3_O_4_@Au@βCD). They found that after β-CD modification, Fe_3_O_4_@Au@βCD nanoparticles have the lowest r2/r1 ratio and can be used as T2 contrast agents in MRI studies. In contrast, Fe_3_O_4_@Au@βCD nanoparticles have good water solubility and biocompatibility and can be selectively taken up by GC cells (MGC-803) and have significant potential in the diagnosis and treatment of GIC [89].

For abdominal magnetic resonance imaging of gastrointestinal tumours, although multifunctional nano-contrast agents have been developed in the laboratory, the first generation of contrast agents are still used in clinical applications. One reason is that some patients with GIC have liver and kidney dysfunction at the same time, and inorganic nano-contrast media can increase the load on liver metabolism. Another reason is that due to the special structure of the gastrointestinal tract, the contrast medium cannot be efficiently retained in tumours site in the gastrointestinal tract. Therefore, the most challenging research direction still consists of improving the retention and targeting efficiency of MRI contrast agents in malignant gastrointestinal tumours by using nanotechnology in the future.

### CT, PET and fluorescence imaging

In addition to nuclear magnetic resonance, CT, PET and fluorescence imaging are also commonly used in clinical observations of malignant tumours. Although the above three imaging methods are not as frequently used as MRI for imaging malignant gastrointestinal tumours, each imaging method has advantages that cannot be replicated by the other three techniques. Here, we briefly introduce the potential of these three imaging techniques when applied to malignant gastrointestinal tumours.

CT, which uses accurate collimated X-ray beams, γ-ray beams, and ultrasonic waves, scans a specific portion of the human body with a highly sensitive detector. It has the characteristics of fast scanning times, low costs, very high spatial resolutions and accurate signal quantization and can be used to examine many diseases [90, 91]. To enhance the retention ability of CT contrast agents in colon cancer, Kimm et al. designed AuNP-conjugated HSP70 monoclonal antibodies that can target mouse colon cancer cells and act as CT contrast agents, showing remarkable imaging capabilities in CT and high sensitivity for single-cell detection [92]. In addition, some studies have found that the use of microneedles and ultrasound can enhance the detection sensitivity of gold nanoclusters and improve the imaging contrast, which has a good effect for identifying GIC [93]. Zhou et al. developed folic acid-coupled silica-coated gold nanoclusters (AuNCs) for use in targeted bimodal fluorescence and CT imaging. The prepared nanoprobe was injected into nude mice via the tail vein after encapsulating silica on the AuNC surfaces and then covalently anchoring folic acid on the AuNC surfaces. The nanoprobe was found to be biocompatible with good targeting properties and exhibited good red fluorescence imaging and CT imaging capabilities [94]. Zhang et al. conducted a clinical study of CT-enhanced scans combined with targeted nanoparticle contrast agents (platelet-derived growth factor receptor-β, Ret and Kit bind to superparamagnetic iron oxide nanoparticles via covalent bonding) for the diagnosis of early gastric cancer. The data show that CT-enhanced scans combined with targeted nanoparticle contrast agents can reveal small nodules in the gastric region. The sensitivity and accuracy of liposome-coated nanoparticle contrast agents combined with CT for early GC diagnosis were improved [95]. In addition, some new CT contrast agents have also been developed in recent years. For example, WS2 nanosheets and oxygen-deficient tungsten oxide WO2.9 nanorods can be used as contrast agents in tumour biologic CT imaging. These new nano-contrast agents can improve the CT imaging effect [96, 97].

**Fig. 5:**
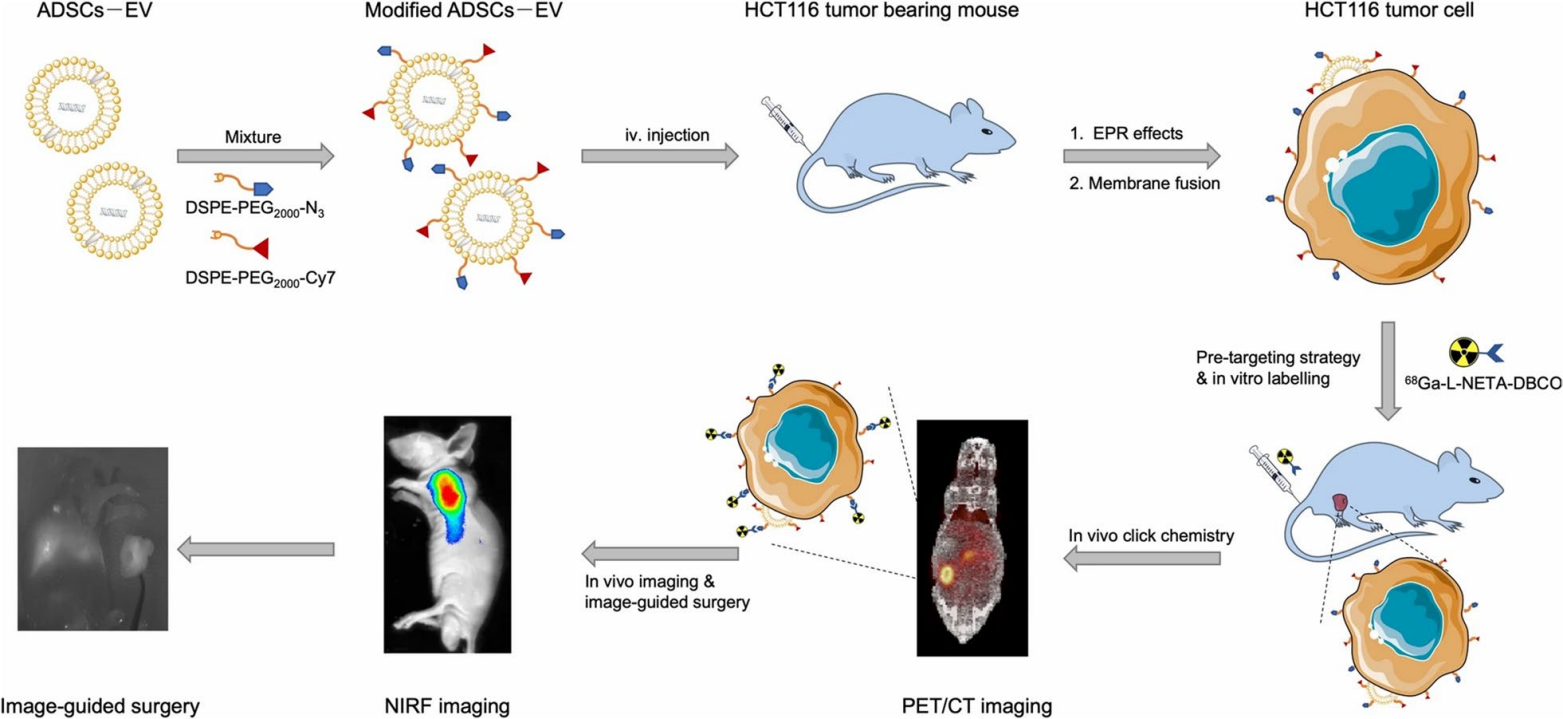
The schematics of multimodal PET and NIRF imaging and real-time NIRF intra-operation based on extracellular vesicles from adipose-derived stem cells [99]

PET is a method that uses highly specific radiopharmaceutical activities to obtain high-quality images for diagnostic purposes. Although the clinical costs are high, it has the advantages of high sensitivity and specificity. For patients with malignant gastrointestinal tumours, the false-positives from PET that are caused by radiopharmaceuticals are a difficult problem that must be overcome in the clinic. [90, 98]. Construction of a nanosized PET contrast media can improve the accuracy of PET imaging and decrease the number of false-positive results. Jing et al. synthesized an extracellular vesicular-based nanoprobe for imaging colon cancer using PET/CT and NIRF imaging and explored its application in image-guided surgery in animal models of colon cancer. The results showed that the extracellular vesicles of adipogenic stem cells had a strong binding ability to tumour cells. The PET images and biological distribution results showed that the optimal pretargeting time of ^68^ Ga-L-Neta-DBCO was 20 h, and the optimal imaging time was 2 h after injection. The NIRF images demonstrated that the tumour images were clear at each time point after the nanoparticles were administered, and the tumour/muscle ratio peaked at 20 h after injection. In addition, both PET/CT and NIRF imaging can clearly show the orthotopic colon cancer model, and real-time NIRF imaging clearly showed the tumour locations and boundaries (Fig. 5) [99]. At present, the nanomaterials used in CT or PET/CT imaging of GIC are still in the preclinical research stage. Multimode combined diagnosis for early diagnosis of gastrointestinal cancer deserves in-depth study. However, in the near future, we are convinced that the advantages of nanotechnology in the field of PET imaging will be widely used in the clinic.

At first, fluorescence imaging was widely used in laboratory scientific research as an imaging method with outstanding advantages such as high sensitivity, fast imaging speed, high security, and low cost. In recent years, the fluorescence imaging reagent, ICG, has been certified by the FDA and has been used in clinical research because of its excellent biosafety, good tissue permeability and low autofluorescence. Most fluorescent contrast agents belong to the hydrophobic structures of small molecules, and the effect in vivo is not as good as that in vitro. Therefore, the in vivo applications of most fluorescent contrast agents need to be coupled to nanoscale drug delivery systems to maximize their functions.[100–102]. Some studies have reported higher UV absorption spectra and stability by nanomodification of ICG while enabling the identification of tumour lymph node metastases at an early stage [103, 104]. Wang et al. synthesized a novel ICG-conjugated gold nanoshell that can effectively accumulate at tumour sites and accurately detect visible and small tumour lesions [105]. In addition, research on the synthesis of near infrared fluorescent nanoparticles and searching for a new method for the diagnosis of gastric carcinoma, nanoparticles or probes combined with tumour targeting ligands to achieve targeting to tumour cells with deeper penetration and a lower background fluorescence signal using NIRF imaging combined with fluorescence endoscopy can be used as an effective tool for the early diagnosis of tumours [106, 107]**.** A recent study by Yoon et al. found that upconversion nanoparticles with emission bands in the range of 650–950 nm could provide deeper tissue imaging [108]. Tian et al. synthesized novel lanthanide-doped upconversion nanoparticles (UCNPs) based on interfacial energy transfer, which exhibited higher tumour-targeting abilities than other conjugates and successfully detected tiny tumours [109]. In summary, although various types of fluorescence imaging nanoplatforms have been used in the study of malignant gastrointestinal tumours, an inherent characteristic of fluorescence imaging technology is that the scattering amplitudes of the fluorescence are inversely proportional to the light wavelength used. As the gastrointestinal tissues and organs in the patient's abdomen are separated by a layer of belly between the light source, the thickness of the fat layer in a patient's abdomen will have a certain impact on the results of fluorescence imaging. With increasing wavelengths, the photon attenuation, tissue fluorescence and scattering mass will decrease significantly, which can easily lead to the inability to excite fluorescent molecules in deep tissue. Therefore, in the future, if we want to expand the application of fluorescence imaging to GIC, penetrating the abdomen and reaching the gastrointestinal tissue with a sufficient ability to activate fluorescent molecules is the main challenge to overcome, and an effective solution may be to modify this method for use in endoscopies [110].

### Conclusion

In this section, we mainly summarize the applications of nanotechnology in the early diagnosis of gastrointestinal cancer. Conventional diagnostic methods, such as endoscopy, tumour marker detection, MRI, CT, PET and NIRF imaging, all have shortcomings, and the introduction of nanotechnology has compensated for these shortcomings. The accuracy and efficiency of early GIC diagnoses were improved. In endoscopy, SERS NPs can increase Raman signal intensities while binding to corresponding targeted ligands, enabling the diagnosis of early tumours and differentiation of diseased tissues. In detections of tumour markers, conventional detection methods have shortcomings such as long detection times, cumbersome processes, difficulties in real-time detection, and vulnerabilities to interference. However, biosensors and QDs based on nanotechnology can improve detection accuracies. Using a multisignal amplification strategy, electrochemical biosensors can achieve highly efficient and sensitive recognition of relevant tumour markers. Using QD-IHC technology, a corresponding antibody probe is prepared, which has more sensitivity and specificity for detecting tumour markers. In biological imaging of tumours, imaging examinations can accurately identify abnormal tissues as advanced tumours. However, for early gastrointestinal tumours, the specificity of conventional imaging examinations decreases. There are still metabolic risks associated with the contrast agents currently used in clinical practice. SLNs, SPOIN, Au NPs and other nanomaterials play important roles in improving tumour imaging, biocompatibility and bioavailability. Therefore, nanotechnology has wide application prospects for early diagnosis of GIC.

At the same time, promoting the clinical use and application of malignant gastrointestinal tumour imaging materials constructed by nanotechnology is the greatest challenge. First, the biological safety, stability and biological distribution of nanomaterials in the human body are still not very clear. In addition, for some nanomaterials, the corresponding detection equipment also needs to be updated and improved in time to maximize the advantages of nanomaterials. The clinical trial and regulatory approval processes before new nanomaterials are tested and rolled out are very slow. Due to the differences in tumour characteristics, tumour accessibility, biological distribution and pharmacokinetics of patients, the success rate of clinical trials is extremely low, hindering the introduction of nanoagents into the market. Therefore, in the future, nanotechnology not only needs to overcome some of its own shortcomings and improve the biocompatibility, effectiveness and diagnostic sensitivity to support detection equipment but also needs to consider its clinical transformation ability and improve its application fields. However, there is no doubt that the in-depth research and development of nanoscience for the prevention, diagnosis and treatment of cancer has indicated that it is very promising approach to overcome the shortcomings of traditional approaches.

## Application of nanotechnology in the treatment of GIC

In the process of clinical diagnosis and treatment of malignant gastrointestinal tumours, accurate diagnostic information can help doctors design the most suitable treatment plans for patients, but in the course of treatment, maximizing the therapeutic effect of the treatment regimen is helpful in prolonging the survival times and prognostic quality of life of patients. At present, the clinical treatment methods used for GIC mainly include surgery, targeted therapy, chemotherapy, immunotherapy or combined therapy. Among them, surgical treatment is the first choice in clinical settings, and the surgical scheme of clinical GIC mainly includes laparoscopic total gastrectomy, laparoscopic distal gastrectomy, laparoscopic subtotal gastrectomy, robot-assisted gastrectomy, robot-assisted laparoscopic gastrectomy, laparoscopic colectomy, robot-assisted laparoscopic colectomy, transanal local excision, transanal endoscopic microsurgery, total mesorectal excision, low anterior resection, and abdominoperineal resection. During GIC surgery, the main challenge consists of accurately and cleanly removing the tumour. In some inoperable areas, because of the unique organ locations and structure of the gastrointestinal tract, removal of tumour lesions by combining photothermal ablation with a gastroscope and with an intestinal endoscope is also a correct and efficient choice. In addition, drug therapies, such as targeted therapy and chemotherapy, are the main treatment option for GIC patients who are unable to have tumour lesions removed by surgery or photothermal ablation. For this type of patient, the greatest clinical challenge is to improve the ability of drugs to accumulate in the tumour area and their efficacy in vivo. Next, we will introduce in detail the applications of nanotechnology in both surgical and drug treatments of malignant gastrointestinal tumours and analyse their future directions.

### Intraoperative imaging and navigation

**Fig. 6:**
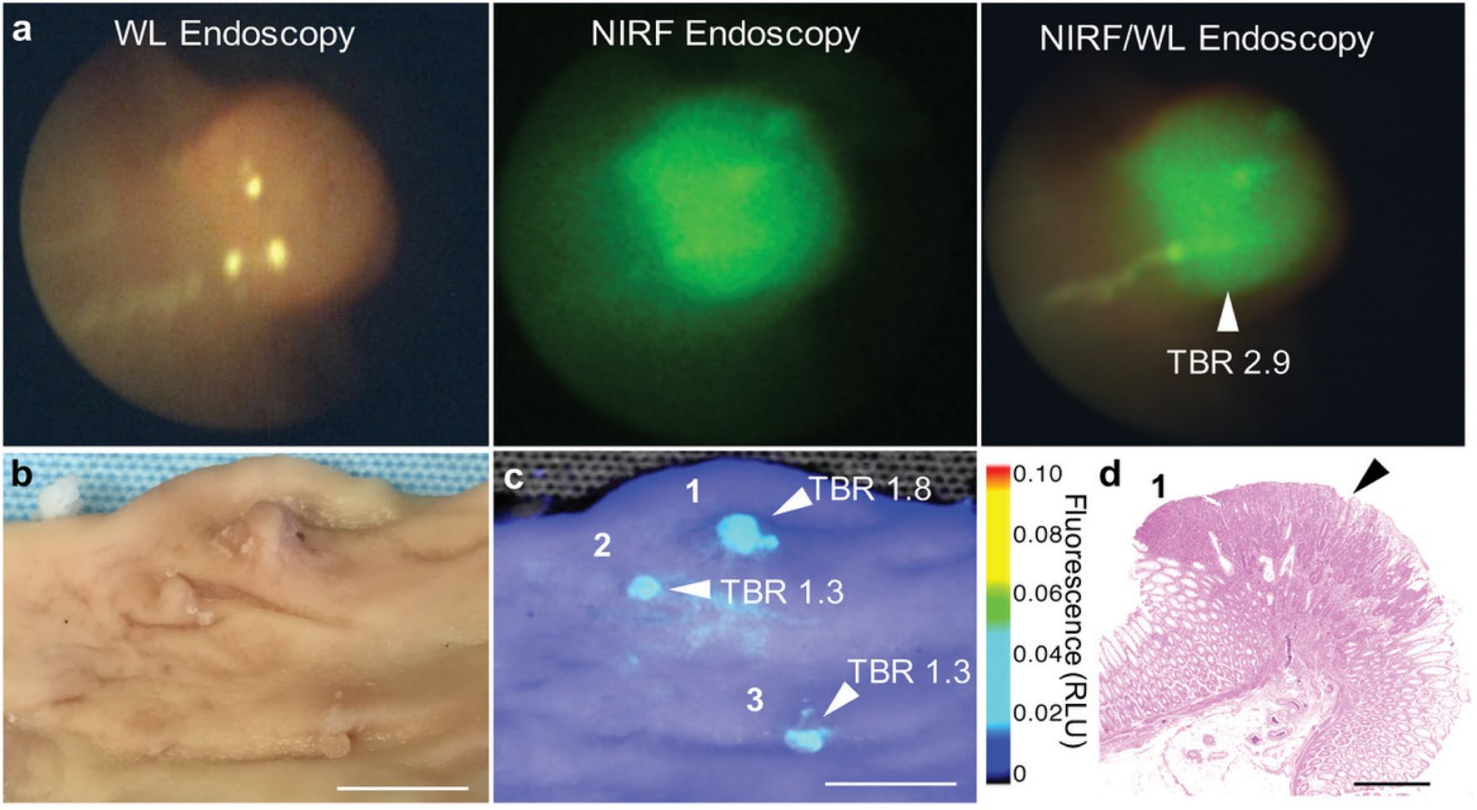
FSN-positive lesions were proved adenomatous polyps during wide-field NIRF imaging in a human-scale model of colorectal carcinogenesis − the APC1311/ + porcine model [114]

Surgical treatment remains the preferred option for resectable GIC. Precise determinations of tumour margins and accurate localization of small tumours are key factors that improve the outcomes of surgical treatments and postoperative quality of life. Therefore, to promote the development of precision medicine, intraoperative imaging technologies, including fluorescence imaging, photoacoustic imaging (PAI), and Raman imaging, have been gradually applied in clinical practice. Compared with free contrast media, the nanometre-sized contrast agents used in the above imaging methods have significant advantages and further improve navigation accuracy during GIC surgery. PAI is an optical imaging technology that can provide strong signals with strong tissue penetration depths and high spatial resolution and can achieve high contrast for soft tissue imaging [111]. Yamada et al. conjugated near-infrared cyanine dye 800RS with PMPC (poly 2-methacryloyloxyethylphosphorylcholine), a polymer with high tumour targeting, to prepare a nanoprobe, 800RS-PMPC, for PA. In a mouse model experiment involving implantation of a colon carcinoma cell line (CT 26), this study obtained high-quality, MRI-consistent, and 3D anatomical imaging PAI results, and the imaging results showed that a 6 × 3 × 3 mm tumour was located at 6 mm subcutaneously. This nanoprobe demonstrates the potential for application in intraoperative tumour development [112]. Fluorescence imaging is also widely used to provide intraoperative imaging navigation for GIC because of its excellent ability to recognize the edges of tumours [113]. Rogalla et al. developed biodegradable near-infrared fluorescent silica nanoparticles (FSNs) and evaluated them using near-infrared fluorescence-assisted white-light endoscopy in an animal model of colorectal cancer. The results showed that FSNs could detect and differentiate adenomas as small as 0.5 mm and had high tumour-to-background ratios, which indicated good clinical prospects (Fig. 6) [114]. Based on biocompatible nanocarrier extracellular vesicles, Jing et al. prepared a novel nanoprobe that can be combined with PET/CT and NIRF imaging to achieve clear visualization of tumour locations and margins for guided surgery. The tumour locations, margins, and residual tumour resections can be clearly observed during mouse tumour resections guided by real-time near-infrared imaging [99].

In addition, SERS and surface-enhanced resonance Raman spectroscopy (SERRS) have become emerging spectral technologies for tumour detection and imaging in recent years. They can achieve high signal tissue imaging based on the characteristic Raman scattering profiles of Raman fingerprints from different tissue molecules [115]. SERS/SERRS NPs are composed of Raman reporter molecules adsorbed on the surface of metal cores (gold or silver NPs), and application of the contrast agent SERRS-NPs can significantly enhance the Raman imaging effect. Enhanced SERS/SERRS signals can be used as tumour markers to guide surgery, and they have high sensitivity and resolution for detecting tumour margins [116]. Harmsen et al. demonstrated that a single dose of SERRS-NPs with high sensitivity could help to effectively detect and delineate small gastrointestinal precancerous lesions in animal models by applying the InVia Raman imaging system, Raman endoscope and Raman/white-light imaging [117].

In summary, the three imaging techniques, namely, PA, fluorescence and Raman spectroscopy all have their own advantages with respect to navigation during GIC surgery. PA imaging combines the high contrast and specificity of spectroscopy with the spatial resolution capability of ultrasound to generate 3D images of biological tissues, and high contrast real-time imaging can be achieved for tissues such as intratumor vascular networks with high absorption coefficients. NIRF has good tissue penetration and a high tumour-background ratio, which has unique advantages in tumour edge recognition and broad application prospects in fluorescence-guided surgery and endoscopy. SERRS can be used for intraoperative tumour imaging and detection of minimal residual lesions based on the characteristics of the Raman fingerprints, and detection of precancerous lesions in the digestive tract can be achieved by SERRS endoscopy. Especially for clinical GIC surgery, the soft tissue deformation-prone nature of the gastrointestinal tract and the difference between intra- and extraluminal views leads to high navigation accuracy in GIC surgery and even requires multiple models to be used at the same time to achieve better resection results. Therefore, preparation of multifunctional contrast agents through nanotechnology is the best choice to provide navigation during GIC surgery in the future. It not only can enhance the signal strengths of the above imaging methods but also can accurately guide and identify small tumours in complex anatomical locations or the lumen when the information changes are caused by real-time stretching caused by soft tissue deformation during gastrointestinal surgery.

### Phototherapy

In the course of clinical GIC treatment, some patients are unable to undergo surgical treatment, or some lesions are not suitable for surgical resection. At this time, due to the availability of gastrointestinal endoscopy and the unique tubular organ structure of the gastrointestinal tract, application of optical therapy in GIC has unique advantages. Phototherapy is a novel tumour treatment with broad prospects and has been studied and used in the gastrointestinal tract [118, 119]. Compared with traditional cancer treatment methods (e.g., surgery, chemotherapy, and radiotherapy), phototherapy has the advantages of high spatiotemporal selectivity, few side effects and low drug resistance[120, 121]. For example, when dealing with early cancer or precancerous lesions in the gastrointestinal tract, phototherapy can effectively remove the lesions by photothermal (PTT) or photodynamic (PDT) means under noninvasive conditions. In the following section, we will mainly discuss multifunctional imaging nanoparticles that use optical methods for the treatment of GIC.

PTT involves irradiation by a laser with a certain wavelength of a photothermal agent that has accumulated in tumour tissue, which generates thermal energy to cause thermal ablation of the tumour cells [122]. When photothermal agents are irradiated with a specific wavelength of light, they absorb the energy of the photons and transform from a ground state monoclinic state to an excited monoclinic state and the increased kinetic energy results in heating of the surrounding microenvironment. As the temperature of the surrounding tissue rises, this increases tissue cell damage and even leads to cell death [123]. Gournaris et al. conjugated gold nanorods (GNRs) with NIRF probes, and dual-modal near-infrared heated and fluorescent gold nanorods (dual-modal GNRs) were synthesized to realize the PTT method of NIRF endoscope image guidance. In a mouse model of transgenic colon cancer, dual-modal GNRs can effectively guide the targeted detection and ablation of abnormal colorectal proliferative lesions [124]. For some nanoparticles that are guided by GIC-specific targets without endoscope guidance, phototherapy of GIC can also be achieved. Shi et al. reported a tumour-targeting and matrix metalloproteinase-2 (MMP-2)-activated nanoprobe (T-MAN) formed by covalently modifying gadolinium-doped CuS micelle nanoparticles with a fluorescent substrate that cRGD and MMP-2 can remove. The activatable nanoprobe enables high spatial resolution MRI and low background fluorescence imaging of gastric tumour cells and lymph node metastases in living mice, with high photothermal conversion efficiency and preferential accumulation in gastric tumours after intravenous injection of the activatable nanoprobe into mice. Their study emphasizes the versatility of tumour-targeting, activable nanoprobes designed using dual biomarker recognition (e.g., αVβ3 and MMP-2) and dual mode imaging (e.g., MRI and NIRF) to improve the selectivity of cancer therapy in vivo [84]. Ye et al. developed a multifunctional nanoplatform to use a combination of gene and photothermal therapy for colon cancer. AuNR@PAMAM-GX1 was constructed by connecting GX1 peptide (cyclic 7 polymer, CGNSNPKSC)-modified polyamide dendrimer (PAMAM, G3) to gold nanorods. AuNR@PAMAM-GX1 was used as a gene delivery vector for PTT and gene therapy in colon cancer cells (HCT-8 cells) [125]. Ni et al. combined MOF (MIL-100) loaded with the chemotherapy drug, mitoxantrone (MTO), with PTT to increase immunogenic cell death. Mil-100 loaded with MTO and hyaluronic acid as nanoparticles (MMH NPs) produced NPs with two therapeutic properties (e.g., photothermal and chemotherapeutic) and dual-imaging modes. Optimal antitumor effects were achieved when MMH NPs were coinjected with anti-OX40 antibodies into colorectal cancer tumours [126]. In addition, other studies have created MOF-based nanodrug delivery platforms to combine chemotherapy and PTT through the guidance of dual imaging modes (e.g., photoacoustic and photothermal) [127]. These studies suggest that targeted delivery of activated enzyme probes into cancer cells could facilitate accurate imaging and on-demand PTT with high spatial and temporal accuracies.

In addition to PTT, photodynamic therapy (PDT) is also a commonly used phototherapy method. PDT is based on the production of reactive oxygen species to induce cytotoxic effects. Upon irradiation, the photosensitiser absorbs photons to transform into an excited electronic state, and the excited single-linear state can undergo intersystem crossover to produce a long-lived excited triplet state and relaxation, with the energy emitted as fluorescence, heat and/or other forms of photophysical energy. The excited triplet state subsequently promotes the production of reactive oxygen species through two mechanisms, which in turn exert cytotoxicity on cancer cells [128–130]. Recently, an effective combination therapy strategy based on porphyrin/camptothecin-fluoroxyuridine triad microbubbles (PCF-MB) has been reported. PCF-MB can be used not only as a contrast agent for ultrasound/fluorescence bimodal imaging but also as a multimodal therapeutic agent in synergistic chemo-photodynamic combination therapy. Guided by ultrasound imaging, local ultrasound exposure can achieve large accumulations of chemotherapy drugs and photosensitizers in tumours, reducing the risk of systemic exposure and chemotherapy resistance [131]. Mao et al. prepared 5-FU-doped SF NPs using cyclic pentapeptide cRGDfk and Ce6 conjugated with regenerated silk fibroin polypeptide (SF) and utilized genipin. The results showed that SF-based multifunctional NPs and PDT could induce high levels of reactive oxygen species and cell death in tumour cells. Combined with PDT, SF-based multifunctional NPs and PDT have ideal tumour targeting activity and significantly decrease tumour loads, and they have good biocompatibility and safety and no obvious toxicity [48]. Yin et al. developed a multifunctional optical/magnetic diagnostic nanoprobe (MNPs-PEG2K-FA@Ce6). Compared with free Ce6 nanoprobes in in vitro studies, MNPs-PEG2K-FA@Ce6 nanoprobes assisted by 633-nm laser irradiation, significantly improved the absorption efficiency of cells and promoted the therapeutic effect of PDT [132]. In recent years, thermally activated delayed fluorescence (TADF) materials have attracted great attention in PDT due to their unique photophysical properties, high structural flexibility and reduced health risks [133]. Fang et al. designed a pure organic photosensitized agent based on TADF nanoparticles for efficient two-photon-excited PDT, which exhibited excellent singlet oxygen production capacity. The antitumor efficiency and biosafety of TADF-based photosensitizers were confirmed in transplanted tumour models, which promoted the potential clinical application of TADF-based pure organic nanomaterials in high-precision bioimaging and high-performance PDT [134].

In summary, phototherapy has unique advantages, such as spatiotemporal selectivity, high efficiency, no drug resistance and noninvasiveness that conventional methods do not possess. Moreover, with the developments in materials science, the combinations of photosensitizers and nanomaterials have overcome the defects of poor penetration, low affinity, low solubility and poor targeting, which thus further highlights the advantages of optical treatments for gastrointestinal tumours. Therefore, we are convinced that the development of optical therapy for use in GIC in the future cannot be separated from the use of nanotechnology.

### Chemotherapy

Chemotherapy is a well-established and routinely used treatment for GIC. Currently, the first-line chemotherapy regimens for GC consist mainly of the application of fluorouracil-based drugs combined with platinum-based drugs or fluorouracil-based drugs combined with taxanes (e.g., docetaxel and paclitaxel). Taxanes, irinotecan and other drugs are second-line recommendations [135]. Third-line treatment regimens, such as trifluridine/tipiracil, are approved by the FDA for clinical use [136]. On the other hand, postoperative adjuvant chemotherapy for colon cancer can be chosen based on the risk factors for recurrence and metastasis. Single-drug fluorouracil chemotherapy (e.g., 5-fluorouracil (5-FU)/leucovorin (LV) intravenous infusion + oral capecitabine) can be used in patients at general risk, while combined chemotherapy using CAPEOX and mFOLFOX6 can be used in high-risk patients, among which single-drug fluorouracil treatments are feasible for patients with mismatch-repair deficiency. For unresectable colon cancer, patients are treated with fluorouracil alone, in combination with oxaliplatin or irinotecan or even with triple-agent combinations, depending on the circumstances. Although a standard course of chemotherapy drugs such as fluorouracil and platinum has significantly improved the survival of patients with gastrointestinal cancer, their inadequate solubility, poor permeability, nonspecific targeting, dose-dependent toxicity to normal tissues, and drug resistance continue to threaten their efficacy [135, 137, 138]. Based on the characteristics and advantages of nanocarriers, nanodrug delivery systems can help achieve efficient and precise targeted drug delivery in the gastrointestinal tract and reduce toxicity and drug resistance. Below, we will review the assistance provided by nanotechnology in the chemotherapy of malignant gastrointestinal tumours in recent years from two aspects: the microenvironmental characteristics of malignant gastrointestinal tumours and the types of nanocarriers with potential for clinical use.

In terms of the tumour microenvironment, the differences in physicochemical properties of different targeted tissue sites, vascular growth and unique hypoxic environments all provide directions for the construction of specific responsive nanocarriers [138]. In addition, multiple enzymes are overexpressed in tumour tissues, such as phospholipase, elastase, matrix metalloproteinases (MMPs), and hyaluronidase (HAase). Therefore, different release mechanisms, such as structural changes in NPs or carrier degradation caused by enzymatic reactions, can achieve effective targeted delivery of therapeutic cancer drugs [139, 140]. Many studies have found that the enzymes overexpressed in gastrointestinal cancers include MMPs; hyaluronidase; cyclo-oxygenase 2; indoleamine 2,3-dioxygenase 1; methionine aminopeptidase-1D; methyltransferase-like 3; pyruvate kinase type M2; and kallikrein-related peptidases [141–148].

The special growth environment of tumour tissue provides a research direction for precise targeted drug delivery and delivery by nanotechnology. The physiological pH of the human circulatory system is approximately 7.4, while the pH of normal tumour tissue is approximately 6.5, which is mainly due to accumulations of lactic acid caused by glycolysis in the hypoxic environment of tumour tissues. However, the pH values of the microenvironments in malignant gastrointestinal tumours are different from those in other tissue sites. The pH in the stomach is approximately 1.5–3.5 and in the colon, it is 6.5 [149, 150]. Therefore, using the pH differences between different parts of the gastrointestinal tract and the emptying times of digestive tract contents, effective targeted drug delivery to tumours in different parts of the gastrointestinal tract can be achieved. Sood et al. prepared chitosan-Eudragit S-100 NPs with pH-responsive release by disulphide bonding thiolated chitosan with thiolated Eudragit S-100, achieving precise targeted drug release for colon cancer [151]. In addition, enzyme reactions can be used to respond to stimuli in nanodrug delivery systems. Sharma et al. developed a therapeutic system, Gal-Dox, targeting β-galactosidase (β-gal), which was preferentially absorbed by HT-29 colon cancer cells through endocytosis mediated by the asialoglycoprotein (ASGP) receptor and activated by elevated lysosomal β-gal enzyme levels, which plays a significant targeted anticancer role [152]. In addition, the glutathione concentrations in malignant gastrointestinal tumours are nearly four times those in normal tissues, so carrier degradation and drug release can be achieved by causing decomposition of the disulphide bonds in nanocarriers. In their study, Feng et al. achieved corelease and significant synergistic antitumour efficacy in colon cancer CT26 cells by co-loading DOX and the immunomodulator, 1-methyl-DL-tryptophan (1MT), into a reduction-reactive polypeptide nanogel, demonstrating the great potential of chemoimmunotherapy for clinical cancer treatment [153]. In addition, the high metabolic efficiency of malignant gastrointestinal tumour cells will also cause their temperatures to be higher (40–42 °C) than the normal physiological temperature (37 °C); cancer cells show more significant negative charges due to increased secretion of lactic acid and increased expressions of anions such as phospholipids [154, 155]. Therefore, some temperature-sensitive or positively charged drug delivery systems are suitable for delivering chemotherapeutic drugs related to malignant gastrointestinal tumours [156]. For example, Carreño et al. achieved sustained local release of a drug with a thermosensitive hydrogel loaded with DOX and demonstrated significant anticancer activity in an in vitro colorectal cancer model [157].

**Fig. 7:**
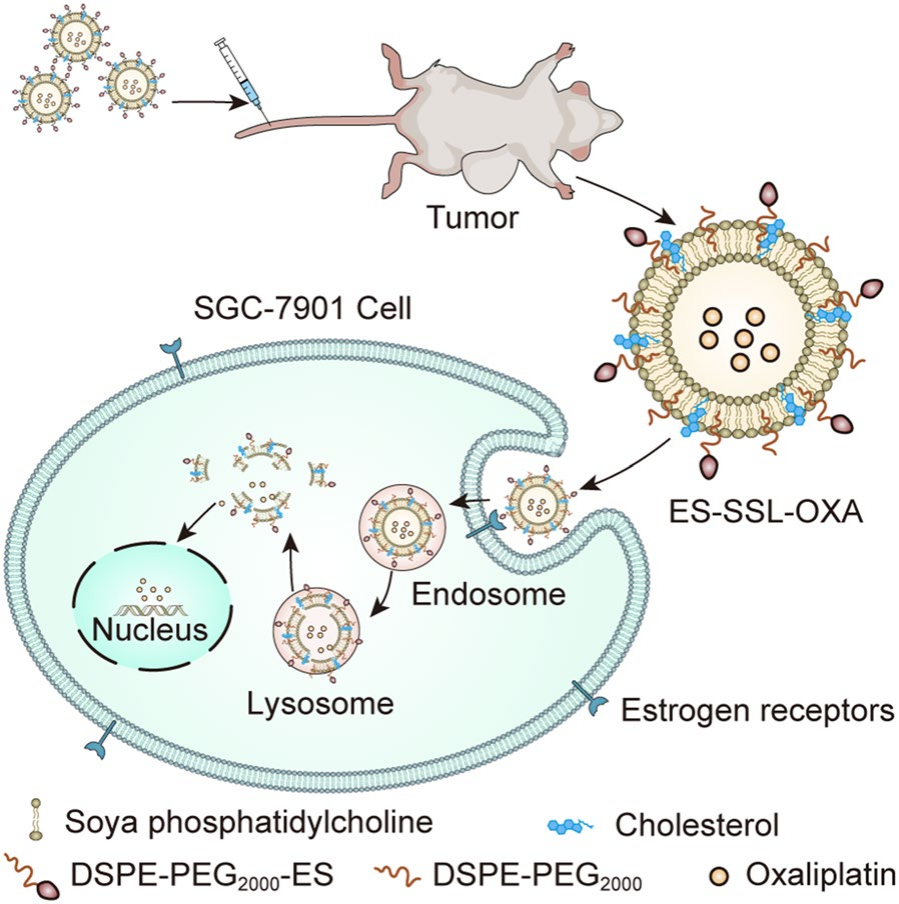
Developed a novel ES-targeted PEGylated liposome for delivery of oxaliplatin to be used to treat estrogen receptor positive gastric cancer [160]

In terms of GIC chemotherapeutic drug delivery carriers, many studies have explored the use of various nanocarriers to load platinum, fluorouracil, taxanes and other chemotherapy drugs to achieve improvements in chemotherapy targeting and drug resistance. FDA-certified nanocarriers, such as liposomes, exosomes, PLGA polymer nanoparticles, nanogels, MOF nanoparticles and other nanocarriers that are moving towards clinical usage, are widely used in the study of delivering chemotherapeutic drugs for GIC. Liposomes are one of the earliest and most widely used nanocarriers with advantages of high biocompatibility, biodegradation and low toxicity and have been used in many research applications, such as chemotherapy drugs, biologics and gene therapy [158, 159]. Sun et al. prepared oestrogen-targeting PEGylated liposomes (ES-SSL-OXA) loaded with oxaliplatin (OXA) and found that ES-SSL-OXA exhibited specific targeting anticancer activity against oestrogen-receptor-positive gastric cancer SGC-7901 cells. It also significantly increased the metabolic times of drugs in blood circulation, increased drug accumulations in tumour sites, and reduced drug toxicity (Fig. 7) [160]. Exosomes are small vesicles containing complex RNA and protein. Exosomes can be used to deliver nucleic acid molecules and anticancer drugs based on their properties and are promising nanodrug carriers [161]. PGM5 antisense RNA 1 (PGM5-AS1) can inhibit the proliferation, migration, and acquired oxaliplatin resistance of colon cancer cells. Therefore, Hui et al. simultaneously loaded pGM5-AS1 and oxaliplatin with engineered exosomes. Further experiments verified that this system could inhibit the proliferation and migration of colon cancer cells and reverse the drug resistance of oxaliplatin [162]. Polymer nanoparticles have high biocompatibility and stability, which can improve the controlled release effect and solubility of drugs [163]. Dos Santos et al. developed 5-Fu-loaded chitosan nanoparticles (CS NPs) and incorporated them into retrograded starch and pectin (RS/P) particles. Based on the specific degradation of RS/P by colon microbiota, this oral drug delivery system has the potential to achieve targeted drug delivery for colon cancer [164]. Fernandes et al. loaded poly(lactic-co-glycolic acid) (PLGA) NPs with 5-FU and paclitaxel (PTX) and functionalized the surface with a monoclonal antibody against sialyl Lewis A (sLeA). The results showed that the encapsulation and modification of PLGA NPs significantly improved the targeted delivery of sLeA antigen (glycochain mediating blood metastasis)-positive gastric cancer cells, reduced biotoxicity and improved the controlled release of drugs [165]. Nanogels are formed by physical or chemical cross-linking of nanoscale hydrophilic polymers. They have the advantages of high loading and delivery capacity, high biocompatibility, easy preparation, controllable particle size, homogeneity and low toxicity and can be used to build pH- and temperature-sensitive nanocarriers [166]. Ohta et al. loaded HA nanogel (HA-CDDP nanogel) coated with cisplatin (CDDP) into an in situ cross-linkable HA hydrogel matrix (HAX), and sustained release of HA nanogel from the HA hydrogel matrix was achieved for more than one week in the treatment of peritoneal disseminated GC. The experimental results showed that this drug delivery system had significant antitumor activity in a mouse model of disseminated peritoneal gastric cancer and significantly decreased the number of peritoneal cancer nodules [167]. Metal–organic frameworks (MOFs) are three-dimensional porous coordination polymers formed by the self-assembly of metal ions and organic ligands, which have the advantages of high porosity, large specific surface area, adjustable pore size, high drug loading, low toxicity and easy chemical modification [168]. Javanbakht et al. loaded 5-Fu in a zinc-based MOF (MOF-5), and a pH-sensitive carboxymethylcellulose (CMC) biopolymer was coated to form biological nanometre hydrogel microspheres, CMC-coated 5-FU@MOF-5 bionanocomposite hydrogel beads (CMC/5-FU@MOF-5). The experimental results showed that CMC/5-FU@MOF-5 achieved colon-targeted controlled release and exerted anticancer activity [169]. In summary, for treating malignant gastrointestinal tumours, the use of chemotherapeutic drugs cannot be improved without the application of nanocarrier technology. In the future, converting increasing numbers of nanocarriers to clinically useful substances will be the main direction of development.

### Targeted therapy

With the discovery of differential gene expression profiles in cancer, individualized targeted therapy for differential gene expressions is gradually emerging. Targeted drugs exert antitumor effects by selectively binding to oncogenic sites and have the advantages of precision, high efficiency and low toxicity. In treating GIC, some patients with special mutations will receive targeted therapy. Human epidermal growth factor receptor 2 (HER2) amplification, epithelial-to-mesenchymal transition amplification, vascular endothelial growth factor (VEGF), fibroblast growth factor receptor 2 amplification/mutation, programmed death ligand 1 (PD-L1) amplification, microsatellite instability (MSI) instability, mismatch repair (MMR) state, and KRAS/BRAF/NRAS gene mutations provide directions for targeted cancer therapy [170, 171]. Although targeted therapy provides precise attacks on cancer cells and can achieve effective anticancer effects, drug resistance, insufficient bioavailability and controlled release effects are still hurdles that need to be overcome. In the following, we will introduce some clinical GIC treatment schemes, as well as the helpful effects of nanotechnology on GIC treatments.

For HER2-positive patients, trastuzumab combined with fluorouracil and cisplatin is often recommended, while fluorouracil combined with cisplatin or oxaliplatin is a conventional first-line chemotherapy regimen for HER2-negative patients [135]. Kubota et al. prepared HER2-targeting gold nanoparticles (T-AuNPs) by combining trastuzumab (Tmab) onto the surface of AuNPs, which demonstrated significant cytotoxicity against Tmab-resistant GC cells by inducing autophagy in GC cells and are a potential new strategy for treating drug-resistant GC [172]. Sabra et al. developed and prepared modified citrus pectin-chitosan nanoparticles (Cet-MCPCNPs) by incorporating cetuximab (Cet) and showed that Cet-MCPCNPs exhibited significant effects in inhibiting cancer cell multiplication, blocking the G2/M phase cell cycle and inducing apoptosis in CRC Caco-2 cells [173]. For those patients with metastatic colorectal cancer who have previously received chemotherapy, antiangiogenic targeted drugs are the main clinical choice. The concentrations of vascular endothelial growth factor are related to increases in tumour invasiveness and decreases in survival rates, which are some of the characteristics of tumour progression. Targeted drugs for VEGFR-2, such as ramucirumab, fruquintinib, sorafenib and apatinib, have been developed, but because of their hydrophobicity, patients need to be given large doses, and high-dose treatments will lead to side effects. To overcome this challenge. Zhang et al. reported a lipid-coated nanodiamond (ND) system loaded with water-insoluble sorafenib (SND). The experimental results show that SND significantly improves the biodistribution, bioavailability and tumour targeting properties of sorafenib. The tumour xenotransplantation model showed significant inhibition of tumour growth and significantly inhibited gastric cancer metastasis to distant organs, such as the liver and kidney [174]. Lin et al. prepared a dual drug carrier, ND-PTX-Cet, using ND loaded with both the microtubule inhibitor paclitaxel (PTX) and Cet, a monoclonal antibody specific for the epidermal growth factor receptor (EGFR). ND-PTX-Cet was shown to induce excessive mitosis and apoptosis in colon cancer cells, resulting in synergistic antitumor activity, as shown by experiments with multiple human colon cancer cell lines (e.g., HCT116, SW620 and RKO) [175]. Among the targeted therapies for CRC, pembrolizumab is indicated as a first-line treatment for patients with unresectable or metastatic CRC with high microsatellite instability (MSI-H) or mismatch repair gene deficiency (dMMR) who have wild-type KRAS, NRAS and BRAF genes. Schmid et al. prepared T-cell-targeted PLGA nanoparticles and loaded them with pembrolizumab, which could be used for targeted delivery of TGFβR1 inhibitors or TLR7/8 agonists, which concentrated immunomodulatory drugs at the site of immunosuppression after systemic administration and significantly inhibited tumour growth and prolonged mouse survival in a tumour-bearing mouse model growing MC38 colon cancer cells [176]. In addition, the hypoxic microenvironment is one of the factors that promotes drug resistance to cancer chemotherapy, and salidroside (Sal), the main active component of Rhodiola rosea, is thought to modulate the hypoxic tumour microenvironment and increase drug resistance. Therefore, in their study, Zhang et al. prepared peptide-functionalized nanoparticles (iVR1-NPs-Apa/Sal) by co-loading Sal with apatinib (Apa) on PLGA NPs and modifying the tumour-homing peptide (iVR1 peptides) on the surface of NPs. In further experiments, it was found that iVR1-NPs-Apa/Sal significantly increased the chemosensitivity of gastric cancer cells to Apa and exhibited significant inhibition of the growth, proliferation, invasion and metastasis of GC cells [177].

**Fig. 8:**
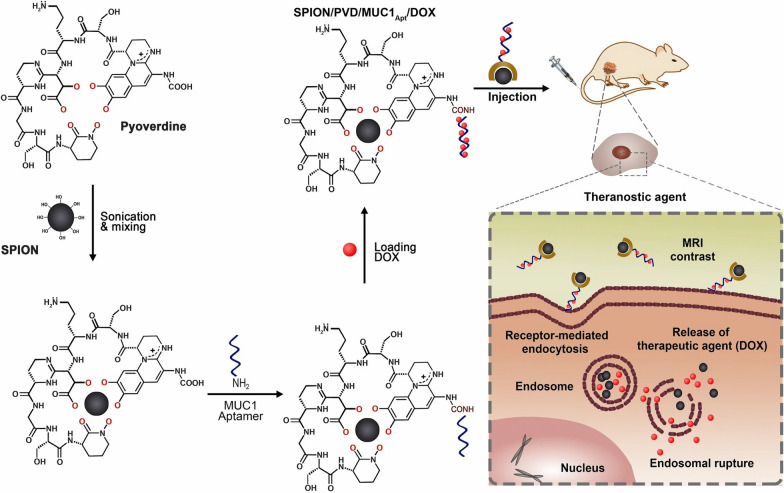
A MUC1Apt-based targeted system for the delivery of DOX-loaded SPION/PVD (SPION/PVD/MUC1Apt/DOX), capable of providing MRI images and preventing cancer cell growth [178]

In the clinical treatment of GIC, the combination of chemotherapy and targeted drugs is a commonly used treatment. Therefore, the efficacy for cancer patients after chemotherapy or targeted therapy is a key clinical concern. Based on the EPR effect of nanomaterials at the tumour site, the traditional segregation of treatment and efficacy assessment can be changed by combining nano-loaded drug-targeted delivery with nanoimaging and visualization technologies to achieve integrated and precise diagnosis and treatment. Nosrati et al. combined superparamagnetic iron oxide nanoparticles-pyoverdine (SPION/PVD) complexes with MUC1 aptamer (MUC1_Apt_). A targeted drug delivery system (SPION/PVD/MUC1_Apt_/DOX) was prepared by loading the chemotherapy drug, doxorubicin (DOX). The results suggest that the targeted agent exhibited significant inhibition of tumour growth, improved survival times, and significantly improved the MRI imaging effect for tumour sites in mice with C26 colon cancer (Fig. 8) [178]. Zhang et al. designed pH/ultrasound dual-responsive, step-targeted, precisely controlled release mesoporous silica-coated gold nanoparticle enteric-soluble particles Au@mSiO2/Ce6/DOX/SLB-FA@CMC (GMCDS-FA@CMC) loaded with chlorin E6 (Ce6) and DOX hydrochloride. GMCDS-FA@CMC can be used in CT image-guided sonodynamic (SDT)-chemotherapy combination therapy and has shown significant targeted anti-colorectal cancer activity in the experimental results [179]. Wu et al. developed sulphur-modified graphene quantum dot NPs with stable fluorescence and loaded them with layered double hydroxides (LDHs) and etoposide (VP16) to create a trifunctional LDH@SGQD-VP16 integrated nanoprobe for simultaneous targeted drug delivery, chemotherapy and fluorescence visualization. The results suggest that this visualization system significantly enhances the pro-apoptotic effect on gastric cancer HGC-27 cells and reduces drug side effects [180].

In summary, the application of nanotechnology in targeted therapies for GIC is nearly the same as that of chemotherapy and can successfully improve the utilization rates of drugs. In addition, some diagnostic nanoparticles provide real-time observation applications to chemotherapy and targeted therapy. In the future, the nanotechnology research field can design nanocarriers with targeting functions according to some unique targets of GIC to further enhance the in vivo utilization of chemotherapeutic drugs or targeted drugs.

### Combination therapy

In recent years, combinations of various treatment schemes have become the clinical treatment trend for GIC. At the same time, advances in nanomaterials have opened up the possibility of multiple combined treatment modalities. PDT combined with PTT, chemotherapy, radiotherapy or immunotherapy can improve the therapeutic effects for gastrointestinal tumours.

**Fig. 9:**
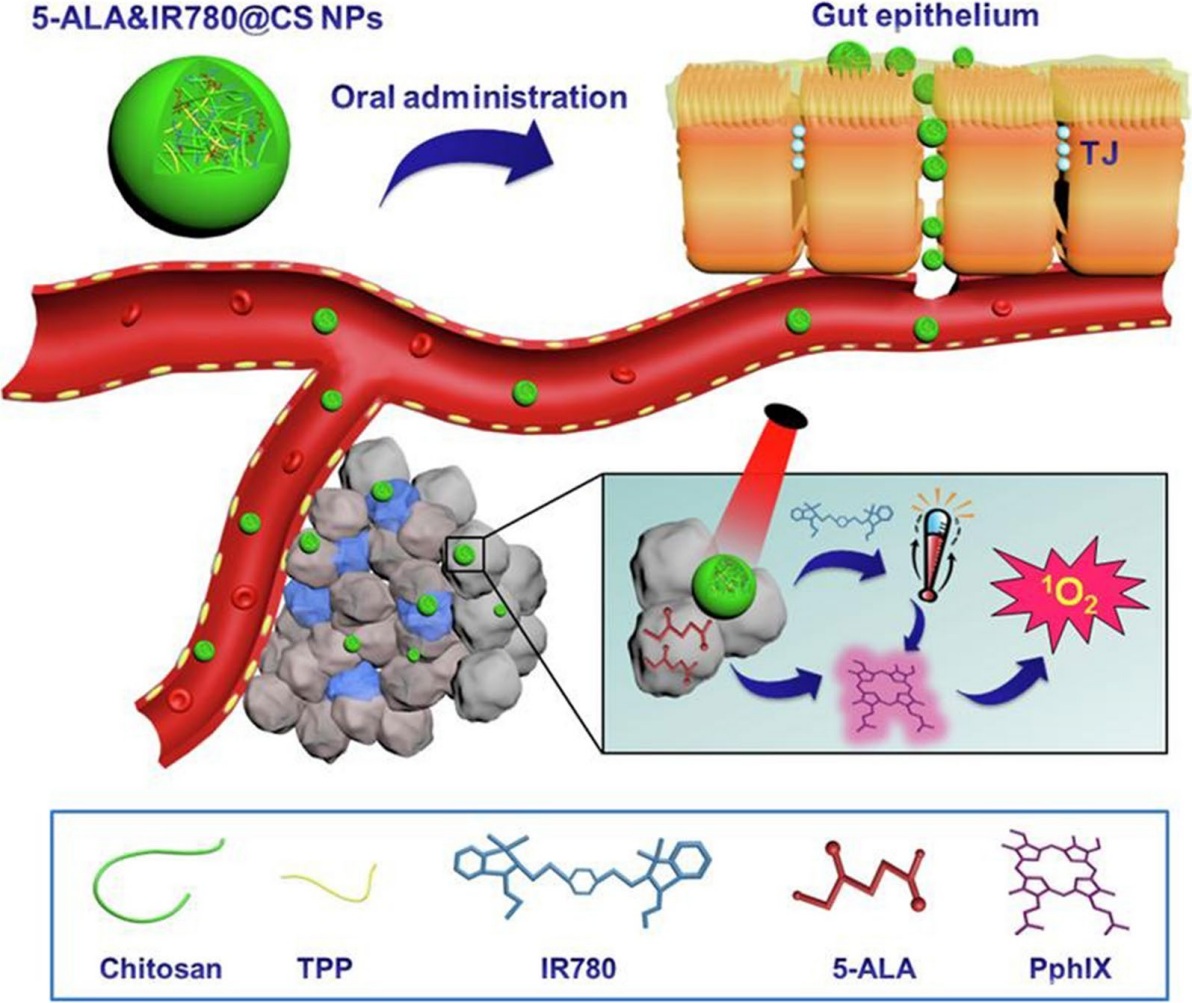
Chitosan NPs encapsulating both photothermal (IR780) and photodynamic 5-ALAreagents lead to photothermally enhanced photodynamic effects [182]

PTT combined with PDT can further improve PDT outcomes by increasing the local permeability of the photosensitizer. Yang et al. developed 20–30 nm SN-38 encapsulated photon micelles for effective cancer therapy. By taking advantage of the supramolecular "π-π" stacking and hydrophobic interaction between SN-38 and unique photonic nanoporphyrin micelles (NPMs), extremely hydrophobic SN-38 was successfully encapsulated in NPM, greatly increasing the water solubility (up to 500 times). At the same drug photosensitizer and light doses, combination therapy with SN-38-encapsulated nanoporphyrin micelles (SN-NPM) increased in vitro antitumor activity by 78- and 350-fold compared with SN-38 and light alone, respectively. NIRF imaging revealed that due to its relatively small size, SN-NPM has an extremely long tumour retention time (> 5 days) and much higher accumulations in tumours than in normal organs. In addition, SN-NPM triple therapy (e.g., PTT, PDT, and chemotherapy) exhibited significantly enhanced antitumor efficacy in vivo for colon cancer compared to monotherapy in nude mice with HT-29 xenografts. Therefore, these sub-100 nm, SN-38-encapsulated photon micelles show great promise in multimodal cancer therapy [181]. Phototherapy can exert its anticancer effects by converting laser radiation energy into hyperthermia or active singlet oxygen. Chen et al. developed CS NPs encapsulating photothermal (IR780) and photodynamic (5-ALA) reagents for photothermal-enhanced photodynamic therapy by using noninvasive oral administration. The results showed that 5-ALA&IR780@CS NPs were stable in acidic conditions similar to the gastric environment, which improved oral absorption of the drug and local accumulations in subcutaneous colonic tumours in mice after oral administration. Furthermore, significant cancer treatment efficacy was observed in vivo after oral administration of 5-ALA&IR780@CS NPs without causing any significant adverse effects (Fig. 9) [182].

For phototherapy combined with radiotherapy and chemotherapy, Gong et al. designed a biomimetic nanoplatform based on hollow polydopamine nanoparticles integrated with platinum NPs to provide radiosensitization under X-ray irradiation in multiple ways, including alleviation of hypoxia, enhancement of tumour apoptosis, and X-ray-induced PDT. This nanoplatform provides an effective new radiosensitization strategy and provides a reference for treating malignant tumours [183]. Triggered liposomes can release genes/drugs in a controlled manner, which can be released in specific regions. However, some release methods, such as pH changes and external heat transfer, have certain limitations. The use of X-ray penetration for liposome triggering provides an alternative and can be used concurrently with radiotherapy. Deng et al. reported X-ray-triggered liposomes containing gold nanoparticles and the photosensitizer Verteporfin. X-ray radiation at 6 MeV induces verteporfin to produce singlet oxygen, which disrupts the stability of the liposome membrane and leads to drug release in the lipid body lumen. The results show that phototherapy combined with radiotherapy and chemotherapy is feasible [184]. 5-Aminolevulinic acid (ALA)-induced PDT based on protoporphyrin IX (PpIX) has been clinically used in the diagnosis and treatment of many cancers, and the combination of effective ALA-PDT and chemotherapy may provide a promising alternative to CRC treatment. Hashemkhani et al. proposed that therapeutic Ag_2_S quantum dots can be optically traced under NIR, conjugated with EGFR targeting Cet, and loaded with ALA for PDT monotherapy or ALA/5-FU for combination therapy to enhance the treatment of EGFR ( +) CRC. The results showed that PDT based on AS-2MPA-ALA-Cet and chemotherapy/PDT combination based on AS-2MPA-ALA-CET-5FU, when combined with strong NIR tracking of nanoparticles, demonstrated that it can achieve a good therapeutic effect on CRC and shows the great potential of multimode tumour synergistic targeted therapy [185].

For phototherapy combined with immunotherapy, considering the adverse reactions and therapeutic efficiency of single immunotherapy for cancer, immunotherapy options range from single-drug therapy to multitechnique combination therapy. At the same time, photodynamically-induced immunogenic death can improve the immunotherapy effect of GIC, so photodynamic therapy combined with checkpoint blocking therapy may provide treatment for extreme malignancies. He et al. designed a nanoscale coordination polymer (NCP) core–shell NP carrying oxaliplatin in the core and a photosensitizer pyrophosphate-lipid adduct (pyrolipid) in the shell (NCP@ pyrolipid) for effective chemotherapy and photodynamic therapy that exploits the synergistic effect between oxaliplatin and pyrolipid-induced PDT to kill tumour cells and trigger an immune response. When combined with anti-PD-L1 therapy, NCP@pyrolipid mediates the regression of light-irradiated primary tumours and unirradiated distal metastatic tumours by inducing a powerful tumour-specific immune response [186]. An immunotherapy based on the natural immune activator, astragaloside III (As), and PDT Ce6 has been developed for colon cancer. It was found that this regimen can effectively activate NK cells and inhibit the proliferation of tumour cells in vitro and can effectively reach tumour sites, induce infiltration of immune cells into the tumour, and enhance the cytotoxicity of natural killer cells and CD8 + T cells in vivo [187]. Yuan et al. reported that PDT therapy mediated by multifunctional NPs loaded with photosensitizer mTHPC (mTHPC@VeC/T-RGD NPs) improved the antitumor efficacy of PD-L1 blockade in CRC. The results showed that mTHPC@VeC/T-RGD NPs under 660-nm NIR laser irradiation can kill tumour cells by inducing apoptosis and necrosis and stimulate a systemic immune response. Under the action of PD-L1 blockade, it can further promote the inhibition of primary tumours and metastatic tumour growth and establish long-term host immune memory to prevent tumour recurrence [188].

In summary, the advantages of combined treatments for diagnosis and treatment of GIC are obviously greater than those of single treatments. For example, PDT and PTT are combined with various imaging tests for CRC to ensure early treatment of patients, thereby reducing the number of cases that require surgery. Image-guided therapies can integrate various pieces of information to infer the distribution and metabolism of PS. In addition, the integration of imaging and therapy into a single imaging-guided, multifunctional cancer treatment platform improves the therapeutic efficiency and safety of the approach. In addition, when phototherapy is used in a synergistic manner with chemotherapeutic agents for GI tumours, it not only reduces resistance to chemotherapeutic agents but also increases the toxicity to GI tumour cells.

### Conclusion

In this section, we mainly summarize the therapeutic effects of nanotechnology in gastrointestinal cancer. Applications of chemotherapy drugs for many patients with GIC result in considerable survival effects; however, poor solubility and bioavailability, systemic side effects, and the development of drug resistance are among the factors that decrease the efficacy of chemotherapy. Therefore, nanotechnology, which enables multifunctional modifications, can undoubtedly provide great assistance for achieving efficient and precise chemotherapy. In addition, the emergence of targeted therapies provides more accurate cancer treatments for patients with gene expression specificity, which significantly improve the survival rates of cancer patients. However, the lack of a controlled release effect and generation of drug resistance are still difficulties to be overcome. Based on the many advantages of the efficient loading and responsive release of nanocarriers, accurate targeted therapy can achieve better treatment effects. Responsive release drug delivery systems based on liposomes, polymer NPs, and metal NPs can significantly decrease the drug doses, toxicities and side effects of chemotherapy drugs and improve the drug resistance of targeted drugs, which have broad prospects for clinical use. However, improving the reproducibility and achieving storage in large-scale production are key challenges that need to be addressed to facilitate the clinical use of nanotherapeutics. On the other hand, timely treatment feedback can effectively improve the effects of cancer treatment, so the combination of imaging technology and precision therapy has become an important development direction for cancer treatment. Currently, the use of nanodrug delivery technology to improve the anticancer effects of chemotherapy, targeted therapy and other therapies and the combination of visualized nanomolecular imaging to help achieve accurate integrated diagnoses and treatments is a new step forward in cancer treatment. Phototherapy has broad application prospects in gastrointestinal cancer based on its advantages of high spatiotemporal selectivity, low invasiveness, low drug resistance and toxicity. The addition of nanotechnology further improves tissue penetration depths, precise targeting and responsive release of phototherapy, which provides accurate treatment for specific cancer tissues, cells and even organelles by PDT, PTT and combination therapy. At the same time, the development of nanophotosensitizers also contributes to the integrated development of gastrointestinal cancer diagnosis and treatment. However, the potential human harms of nanophotosensitizers still need to be effectively addressed to advance their clinical translation and application. In conclusion, nanotherapies, which have great promise, still must overcome many hurdles before being used in universal clinical applications.

## Prospects

GIC is a global health problem, the incidence of which is gradually increasing at younger ages. The survival rates for GIC are significantly lower after metastasis, so it is necessary to strengthen monitoring and apply active treatment methods. However, the current diagnoses and treatments of gastric and colorectal cancer are not satisfactory. Therefore, there is an urgent need for highly specific and precise diagnostic techniques combined with safer and more effective treatment strategies. Nanotechnology has great potential in the diagnosis and treatment of gastrointestinal tumours and is worthy of further exploration. This review introduces the applications of nanotechnology for the diagnosis and treatment of GIC. For diagnosis, endoscopic diagnoses based on SERS nanoparticles can help us distinguish normal and precancerous tissues, identify missing lesions, and improve the sensitivity of early GIC diagnosis; AnNPS, SiNWs and QDs can increase the diagnostic sensitivity of tumour markers in serum or tissue samples of GIC, which is of reference significance for developing a noninvasive and precise clinical tool for cancer detection and screening. In MRI/CT/PET and NIRF imaging, nanoparticles (e.g., SLNs, SPOIN, and AuNPs), nanoprobes and imaging systems based on nanotechnology have good targeting and biocompatibility, which can improve the efficiency of tumour imaging and early diagnosis. In the treatment of GIC, nanoparticles are widely used in drug delivery targeting therapy, phototherapy and surgical navigation because of their excellent properties. Nanodrug delivery systems improve the biocompatibility of drugs and improve the efficiency of tumour treatment; the application of PDT/PTT enriches the treatment of GIC, and PDT/PTT combined with other therapies is of great significance for inhibition. Nanotechnology is also of great significance in surgical navigation. In preoperative or intraoperative interventions of tumours (such as injections of fluorescent nanocontrast agents) and detection, we can clearly find the locations and edges of tumours for accurate resection. Overall, nanotechnology plays a crucial role in diagnosing and treating GIC (Table 1).

**Table 1:** Application of nanotechnology in early diagnosis and comprehensive treatment of gastrointestinal cancer

	Approaches	Advantages	Limitations	Nanotechnology	Joint nanotechnology	References
Diagnosis	Endoscope	Convenient and efficient;Direct observation	Poor identification of small lesions; Strong subjectivity	SERS NPs Capsule endoscopy Confocal laser microendoscopy	Improve sensitive differentiation of small and other difficult-to-detect lesions Highly targeted	[56–67]
	Tumour Markers	Convenient, high clinical value,	Limited level of expression, Susceptible to interference, Invasive	Nanobiosensors, SiNWs, SiNW-FETs, QDs	1. Higher accuracy and sensitivity of detection 2. Easier operation 3. Noninvasive	[73–80]
	MRI	High soft tissue contrast and no ionising radiation	Nonspecific, Rapid clearance, Tissue deposition	Gd-Liposomes and Gd- nanocomplexes, SPOIN	1, High biocompatibility 2, Highly targeted 3, Higher detection accuracy and sensitivity	[81–89]
	CT	Fast scanning time, Lower cost, High spatial resolution	Limited soft tissue identification, Low contrast accumulation	AuNPs/GNRs, AuNCs, SPOIN, WS2 nanosheets, WO_2.9_ nanorods	1, Enhanced accuracy and sensitivity 2, Remarkable imaging effect 3, No toxicity 4, Multimodal imaging	[93–97]
	PET	High sensitivity and specificity; Easy to find metastatic lesions	High costs False positives in patients with inflammatory conditions	Dendritic macromolecular systems and extracellular vesicular nanoprobes	1. Reduce false positives 2. Non-toxic 3. Highly targeted	[99]
	Fluorescence imaging	Fast imaging, High sensitivity	With longer wavelength, the quality of tissue fluorescence and scattering decreases	ICG-Liposomes; Upconversion nanoparticles	1. Increased diagnostic accuracy and sensitivity 2. High optical stability 3. No toxicity	[106–109]
Treatment	Intraoperative navigation and surgery	Minimally invasive	Poor localization of tumour margins and tumours	ICG-SPION Cluster, NIRF with endoscopy, SERS/SERRS NPs	1. Increased sensitivity and resolution at tumour margins 2. High tissue penetration	[99, 112–117]
	Chemotherapy	Good therapeutic effect	Low solubility, poor permeability, Non-specific targeting, Dose-dependent toxicity	Liposomal, Albumin, CS NPs, PLGA NPs, Nanogel, MOF	1. Increased effectiveness of chemotherapy 2. Less toxic side effects of chemotherapy 3.Highly targeted	[151–157, 160–169]
	Targeted therapy	Well-targeted and low toxic side effects	Drug resistance, Insufficient bioavailability, Insufficient controlled release	AuNPs, ND, PLGA NPs SPION/PVD; Graphene quantum dots	1. Improving the bioavailability of delivered drugs 2. Higher targeting performance 3. Targeted controlled release	[172–180]
	Phototherapy	High temporal selectivity and low side effects and low drug resistance	Low photothermal conversion efficiency, irradiation depth and irradiation accuracy	NIRF probe bound gold nanorods and nanoporphyrin micelles;	1. Higher photothermal conversion efficiency 2. Highly targeted 3. Combination of multiple treatment modalities	[124–127, 131–134]
	Combination therapy	High treatment efficiency and Providing access to treatment for extreme malignancies		Trigger liposomes, AuNPs, Core–shell nanoparticles, Nanoporphyrin micelles;	1、High treatment efficiency 2、providing access to treatment for extreme malignancies	[181–185]

*SERS* Surface-enhanced raman scattering; *NP* Nanoparticles; *SiNWs* Silicon nanowires; *SiNWFETs* Silicon nanowire field-effect transistors; *QDs* Quantum dots; *SPOIN* Superparamagnetic iron oxide nanoparticles; *AuNPs* Gold nanoparticles; *GNRs* Gold nanorods; *AuNCs* Gold nanocluster; ICG Indocyanine green; *SERRS* Surface-enhanced resonance raman spectroscopy; *NIRF* Near infrared fluorescence; *CS* Chitosan; *MOF* Metal-organicframeworks; *PLGA* poly lactic-co-glycolic acid; *ND* Nanodiamond; *PVD* Pyoverdine

Nanotechnology has shown its potential in early screening for GIC, biological imaging, targeted therapy, surgical navigation and other aspects. However, very few nanomaterials are currently used in clinical diagnosis and treatment. The main reasons include the low transfer efficiency of nanoparticles to tumours, neglected cytotoxicity and long-term clearance of ions, lack of understanding of the molecular mechanism of ions acting on living cells, and technical challenges such as synthesis and excellent performance of nanoparticles [189, 190]. Meanwhile, due to the great heterogeneity among human diseases and basic experimental models, laboratory research cannot be immediately transformed into human clinical research. Moreover, all nanoparticles must be carefully evaluated before being used in clinical trials to prevent these multifunctional and complex nanoparticles from exerting unexpected effects on the human body. In the future, we believe that a comprehensive understanding of the metabolic pathways of nanomaterials in the human body, the interactions among nanomaterials and tumours or other organs, and the relationships between the size, charge and cytotoxicity of nanomaterials are helpful to promote the clinical use of nanotechnology in GIC and improve the clinical therapeutic effects for GIC.

## Data Availability

Not applicable.

## Abbrevations

5-FU: 5-Fluorouracil
AuNPs: Gold nanoparticles
AuNCs: Gold nanocluster
ALA: Aminolevulinic acid
Ce6: Chlorin E6
Cet: Cetuximab
CMC: Carboxymethylcellulose
CDDP: Cisplatin;HA:hyaluronan
CS NPs: Chitosan nanoparticles
CRC: Colorectal cancer
CT: Computed tomography
ctDNA: Circulating tumour DNA
CTCs: Circulating tumour cells
DTPA: Diethylenetriaminepentaacetic acid dihydroxide
DOX: Doxorubicin
EPR: Enhanced permeability and retention effect
ES-SSL: Estrogen-targeting PEGylated liposomes
FITC: Fluorescein octadecylamine isothiocyanate
FSN: Fluorescent silica nanoparticles
GIC: Gastrointestinal cancer
GC: Gastric cancer
GNRs: Gold nanorods
Gd: Gadolinium
HAX: Hydrogel matrix
IHC: : Immunohistochemistry
ICC: Immunocytochemistry
ICG: Indocyanine green
LV: Leucovorin
LDHs: Layered double hydroxides
MRI: Magnetic resonance imaging
MSI: Microsatellite instability
MWCNTs: Carboxylated multiwalled carbon nanotubes
MOF: Metal-organicframeworks
MCPCNPs: Modified citrus pectin-chitosan nanoparticles
MMP-2: Matrix metalloproteinase-2
MMR: Mismatch repair
MUC1Apt: Mucin 1 aptamer
MTO: Mitoxantrone
MiRNA: MicroRNA
NPs: Nanoparticles
NIR: Near infrared ray
NIRF: Near infrared fluorescence
NMR: Nuclear magnetic resonance
NOTA: 1, 4, 7-Triazacyclonane-1, 4, 7-Triacetic acid
ND: Nanodiamond
EGFR: Epidermal growth factor receptor
NPM: Nanoporphyrin micelles
NCP: Nanoscale coordination polymer
OXA: Oxaliplatin
PET: Positron emission tomography
PDT: Photodynamic therapy
PTT: Photothermal therapy
PTH: Poly thionine
PAI: Photoacoustic imaging
PMPC: Poly 2-methacryloyloxyethylphosphorylcholine
PLGA: Poly(lactic-co-glycolic acid);
PTX: Paclitaxel
PD-L1: Programmed death ligand 1
PVD: Pyoverdine PCF-MB:Porphyrin/camptothecin-fluoroxyuridine triad microbubbles
PpIX: Protoporphyrin IX
QDs: Quantum dots
RS/P: Retrograded starch and pectin
SERS: Surface-enhanced raman scattering
ssDNA1: Single-stranded DNA1
SiNWs: Silicon nanowires
SiNWFETs: Silicon nanowire field-effect transistors
SLNs: Solid lipid nanoparticles
SPION: Superparamagnetic iron oxide nanoparticles
SERRS: Surface-enhanced resonance raman spectroscopy
SND: Sorafenib loaded nanodiamond
Sal: Salidroside
SDT: Sonodynamic
SF: Silk fibroin polypeptide
TH: Thionine
TMGNs: 99MTc-labeled methionine-gold nanoparticles
TADF: Thermally activated delayed fluorescence
VEGF: Vascular endothelial growth factor
VP16: Etoposide
